# Magnetic ionic liquids: synthesis, properties and applications

**DOI:** 10.1039/d6ra09468f

**Published:** 2026-11-10

**Authors:** Harvinder Singh Sohal, Navneet Kaur, Navdeep Kaur, Manvinder Kaur, Haesook Han, Pradip K. Bhowmik, Neeraj Bala

**Affiliations:** a Materials and Natural Products Laboratory, Department of Chemistry, Chandigarh University Mohali Punjab 140413 India drharvinder.cu@gmail.com; b Department of Chemistry, Sri Guru Granth Sahib World University Fatehgarh Sahib-140407 Punjab India; c Chitkara School of Planning and Architecture, Chitkara University Rajpura-140401 Punjab India; d Department of Chemistry and Biochemistry, University of Nevada Las Vegas Las Vegas Nevada 89154 USA pradip.bhowmik@unlv.edu; e Department of Chemistry, Patel Memorial National College Rajpura-140401 Punjab India neerajpmn@gmail.com

## Abstract

By combining the tunability and little volatility of ionic liquids with magnetic responsiveness, magnetic ionic liquids (MILs) have become multipurpose materials that provide easy magnetic manipulation, separation, and recovery. With a focus on the connections between molecular structure, magnetic response, viscosity, transport properties, stability, and functional performance, this review critically explores recent developments in the design, synthesis, physicochemical properties, and applications of MILs. For use in catalysis, extraction, separation, environmental remediation, sensing, energy, and smart materials, recent advancements such as metal–ligand engineering, bimetallic MILs, sol–gel immobilization, microwave-assisted synthesis, and magnetic nanoparticle-based hybrids are highlighted. In addition to the frequently disregarded problems of metal leaching, toxicity, recyclability, cost, and scalability, special emphasis is paid to the ongoing trade-off between magnetic susceptibility and viscosity. The review also identifies two new methods for creating task-specific MILs with optimum properties: computational modeling and machine learning-assisted design. Overall, this review shows how MILs have evolved from proof-of-concept magnetic liquids to functioning platforms that are technologically relevant, rationally constructed, recoverable, and sustainable.

## Abstract 2

By combining the tunability and little volatility of ionic liquids with magnetic responsiveness, magnetic ionic liquids (MILs) have become multipurpose materials that provide easy magnetic manipulation, separation, and recovery.

## Graphical abstract

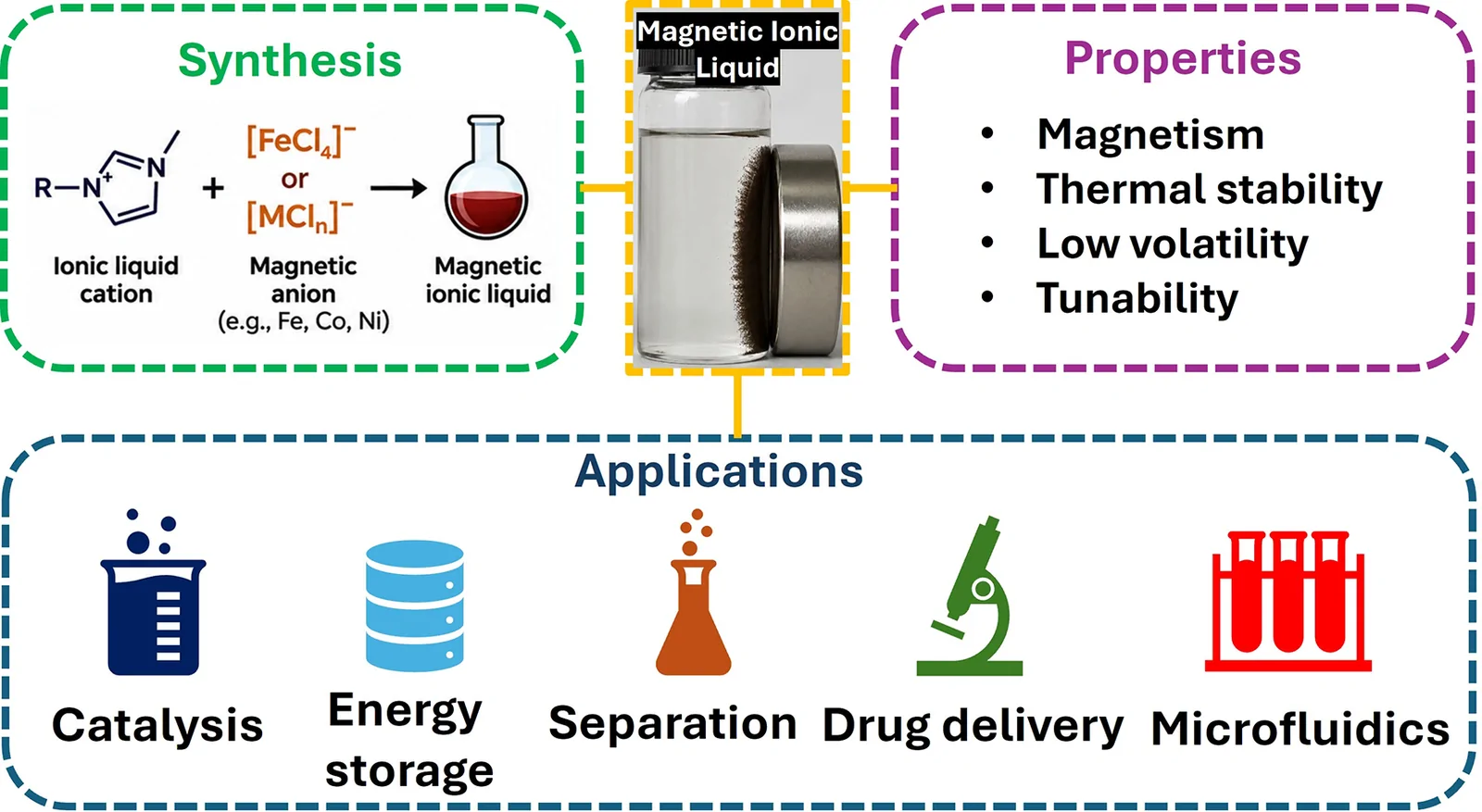

## 1. Introduction

Magnetic ionic liquids (MILs) are a specialized class of ionic liquids (ILs), conventionally defined as salts with melting points below 100 °C, that exhibit magnetic-field responsiveness, typically arising from paramagnetic species.^1^ Unlike conventional ferrofluids containing dispersed magnetic nanoparticles, MILs incorporate magnetic metal-containing ions or coordination complexes into the cation, anion, or both. Common magnetic centers include transition-metal ions (*e.g.*, Fe^3+/2+^, Co^2+^, and Mn^2+^) and lanthanide ions (*e.g.*, Gd^3+^ and Dy^3+^).^2^

While reported saturation magnetization can range from <1 to multiple emu g^−1^, MILs can display magnetic susceptibilities ranging from roughly 10^−1^ to 10^−2^ emu g^−1^ Oe^−1^, depending on the magnetic center, coordination environment, concentration, and anion structure. As a result, magnetic characteristics can be added to the liquid without creating a distinct solid magnetic phase.^3^

Compared to traditional magnetic nanoparticle suspensions, which may experience aggregation, sedimentation, oxidation, and phase separation, integrating magnetic functionality into ILs provides advantages.^4^ MILs provide single-phase, magnetically sensitive liquids that allow for magnetic manipulation, separation, and recovery without the need for further filtration or centrifugation since magnetic species are integrated directly into the ionic structure.^5^ On the other hand, metal integration can impact conductivity and thermal stability while increasing viscosity, density, and cost. Depending on composition and conditions, MIL densities can reach 1.2–2.0 g cm^−3^, while viscosities can range from ten to thousands mPa s. Magnetic responsiveness, viscosity, conductivity, stability, cost, toxicity, and recyclability must all be balanced in MIL design.^6,7^

Combining several functions into a single molecular platform is another benefit of MILs.^8^ Properties including hydrophilicity/hydrophobicity, acidity/basicity, metal-ion affinity, gas solubility, viscosity, conductivity, and magnetic susceptibility can all be simultaneously adjusted by carefully choosing the cation, anion, functional group, and magnetic center.^9^ For instance, transition-metal or lanthanide centers offer magnetic responsiveness, and functional groups like –NH_2_, –COOH, –SO_3_H, ether, phosphonate, or fluorinated moieties can be added to improve selective interactions with target molecules.^10^ This structural flexibility sets MILs apart from traditional magnetic fluids, where dispersed nanoparticles are the main source of magnetic functionality.

Despite these benefits, there are still a number of obstacles in the way of MILs development. Many MILs need expensive precursors, metal-containing reagents, moisture-controlled conditions, or multistep synthesis, all of which can raise manufacturing costs and restrict scalability.^11^ Concerns about toxicity, environmental persistence, and end-of-life disposal may also be raised by the presence of transition metals or fluorinated anions. Additionally, variations in magnetic-field intensity, measurement temperature, water content, purity, viscosity, and magnetic-property characterization techniques make it difficult to compare reported MILs.^12^ As a result, direct comparisons of stated magnetic susceptibility or magnetization levels are not always possible.^4^ Transforming MILs from laboratory-scale materials into useful technologies thus requires a thorough evaluation of structure–magnetism–property connections, repeatability, recyclability, and application-level performance.^8^

The synthesis, magnetic behavior, and specific uses of MILs have already been reviewed in a number of publications;^13^ however, many of these reviews have mainly focused on synthetic techniques, basic magnetic properties, or specific application areas.^2^ Given the quick development of multifunctional and task-specific MILs,^14^ a thorough assessment linking molecular design, synthesis, physicochemical properties, magnetic response, application performance, and practical limits is still crucial.^15^

Unlike previous reviews that primarily emphasize MIL synthesis, fundamental magnetic properties, or specific applications, this review provides an integrated critical assessment linking molecular design, structure–property relationships, magnetic behavior, and application-level performance.^16^ The literature reviewed spans publications from 1972 to 2026, with a primary focus on recent advances in magnetic ionic liquids. Approximately 74% of the cited literature was published during 2021–2026, 19.8% during 2011–2020, and 6.2% during 1972–2010, highlighting the emphasis on recent developments while retaining key foundational studies. It further identifies key research gaps in standardization, reproducibility, sustainability, scalability, and recyclability, while comparing the scope of existing reviews to establish the distinctive contribution of the present work.

## 2. Synthesis of magnetic ionic liquids

### 2.1. Material-based synthesis of magnetic ionic liquids

Magnetic susceptibility, viscosity, conductivity, thermal stability, and chemical compatibility are all determined by the choice of magnetic ion, ionic building blocks, ligand environment, and cation–anion combination in the synthesis of MILs.^14^ Fe^3+^, Co^2+^, Mn^2+^, Ni^2+^, and lanthanide ions are examples of paramagnetic centers that can be added to either the cationic or anionic component.^17^ Significantly, structural alteration of either ionic component offers a way to regulate the physicochemical characteristics of the resultant MILs while adjusting the magnetic response. The five-generation classification is based primarily on changes in molecular design strategies and functional capabilities, rather than a strict chronological sequence of publications. Each generation represents a dominant design emphasis, from single magnetic centers and coordination engineering to functionalization, dual-paramagnetic systems, and multifunctionality, while emerging metal-free and computationally guided approaches reflect ongoing developments that may coexist with earlier strategies.

#### 2.1.1. First generation: incorporation of a single magnetic ion

Tetrachloroferrate (iii), [FeCl_4_]^−^, was one of the most extensively studied magnetic anions in the first generation of MILs, which mainly entailed the inclusion of a single paramagnetic metal center.^18^ Eight [FeCl_4_]^−^-based MILs with various organic cations were synthesized by Zafar *et al.*, who showed that cation alteration significantly affected their electrochemical and magnetic characteristics. These MILs exhibited an effective magnetic moment (*µ*_eff_) of approximately 5.66 µB. Separately, the reported *χ*_MT_ values ranged from 3.49 to 4.50 emu mol^−1^ K Oe^−1^, consistent with the magnetic behavior expected for high-spin Fe^3+^. The effective magnetic moment and *χ*_MT_ represent distinct magnetic quantities; therefore, their numerical values should be interpreted separately rather than compared directly. Their heterogeneous electron-transfer rate constants reached 3.60 × 10^−3^ cm s^−1^, and their diffusion coefficients were roughly 7.55 × 10^−1^ cm^2^ s^−1^, indicating that magnetic inclusion can be accomplished without sacrificing beneficial electrochemical transport features.^19^

Later research extended single-metal MIL chemistry to lanthanide-containing systems, where stronger paramagnetic responses are provided by the greater number of unpaired electrons in ions like Gd^3+^. The identity and concentration of the metal center directly determine magnetic susceptibility, as demonstrated by Knoop *et al.*'s quantitative evaluation of the magnetic characteristics of lanthanide-containing paramagnetic ILs using Evans NMR.^20^

Overcoming the trade-off between fluidity and magnetic response has been the subject of more recent studies. In order to achieve significantly lower viscosity while maintaining high magnetic susceptibility, Abbasi *et al.* created MILs with functionalized diglycolic acid ester ligands and rare-earth/transition-metal centers.^16^ In another work, Abbasi *et al.* observed effective magnetic moments of 2.78–5.16 µB, viscosities as low as 198.3 cP at 22.8 °C, and thermal stability up to 345 °C for certain diglycolamide MILs using transition-metal chelates with [NTf_2_]^−^-based systems. A significant advance in synthetic simplicity and practical handling was demonstrated by the fact that certain MILs could be produced *in situ* in water in less than 30 seconds, and some formulations remained chemically stable in aquatic conditions for longer than six months.^21^

These results show that first-generation MIL design has advanced from merely adding a magnetic ion to managing the ionic architecture and metal–ligand environment. This broader optimization does not, however, make the first generation mutually exclusive from later design strategies; it describes the dominant design emphasis in this classification. Therefore, when creating application-specific MILs, the balance among magnetization, viscosity, conductivity, and stability must be taken into account rather than simply increasing the concentration of magnetic centers to get the ideal material. Subsequent generations with numerous magnetic centers, functionalized ligands, and multifunctional cationic/anionic architectures are based on this progression.^22^

#### 2.1.2. Second generation: coordination environments and magnetic ion diversity

The realization that adding a single paramagnetic ion is insufficient to maximize magnetic performance gave rise to the second generation of MILs.^18^ Rather, magnetic susceptibility can be adjusted together with viscosity, thermal stability, and chemical functionality by varying metal identity, oxidation state, coordination number, ligand field, shape, and the location of magnetic centers within the cation or anion. As a result, lanthanides like Gd^3+^, Dy^3+^, Ho^3+^, Er^3+^, and Tb^3+^ and transition metals like Co^2+^, Ni^2+^, Mn^2+^, and Fe^3+^ have been added to various coordination settings. The design space of MILs has significantly expanded beyond traditional [FeCl_4_]^−^ systems as a result of this diversification.^23^

A particularly important advance was reported by Abbasi *et al.*,^24^ who synthesized 30 MILs containing lanthanide and transition-metal centres coordinated with straight-chain and branched diglycolamide ligands. The resulting materials exhibited reported effective magnetic moments ranging from 4.71 to 21.08 µB. These values describe the effective magnetic moment and should be compared with values reported for the same parameter under comparable measurement and calculation conditions.

Systems comprising transition metals showed thermal stability up to 235 °C, while the comparable lanthanide systems showed thermal stability at about 192 °C. It is interesting to note that substituting a transition metal for a lanthanide center in either the cation or the anion could simultaneously improve thermal stability and reduce viscosity, proving that metal selection influences more than just magnetic response.^24^

Knoop *et al.* created a series of lanthanide-containing PILs, [C_*n*_C_1_Im]_3_[MCl_3_X_3_] (*n* = 4, 6, 8; M = Gd, Dy, Ho; X = Cl or Br), further demonstrating the function of the magnetic ion (Fig. 1). The strongest magnetic susceptibilities for mononuclear paramagnetic ILs at the time were found in systems comprising Dy and Ho, which significantly outperformed similar Fe-based systems. Additionally, the study showed that Evans NMR could quantitatively quantify the magnetic response while taking water content into account. This is crucial since hygroscopicity can significantly increase the uncertainty of magnetic-property measurements.^20^

**Fig. 1:**
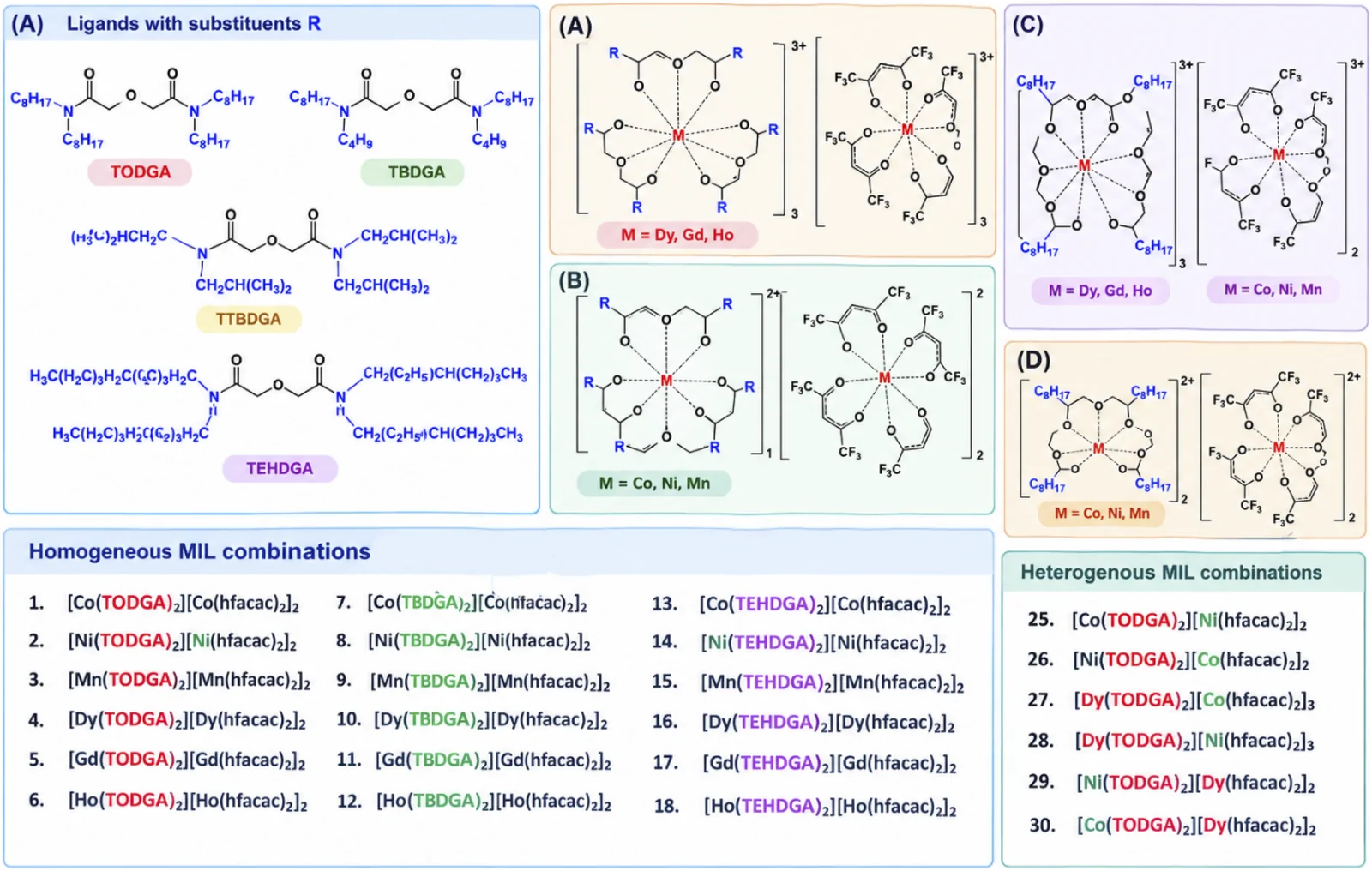
Chemical structures and numbering scheme of homogeneous and heterogeneous MILs: (A) lanthanide-based, (B) transition-metal-based, (C) lanthanide-cation/transition-metal-anion, and (D) transition-metal-cation/rare-earth-metal-anion MILs.^20^

MIL characteristics can also be controlled by coordination chemistry. Effective magnetic moments of 2.78–5.16 µB were observed in recent transition-metal MILs based on Co^2+^ and Ni^2+^ with [NTf_2_]^−^ counterions. For instance, the effective magnetic moment rose from roughly 2.78 to 4.47 µB when the metal was changed from Ni^2+^ to Co^2+^, but only slight changes (2.78–2.81 µB) were obtained when the ligand structure surrounding Ni^2+^ was changed. This suggests that when the coordination environment stays in the same spin state, metal identity can have a stronger influence on magnetic behavior than ligand substitution.^21^

The second-generation MILs show that simultaneous control over magnetism, viscosity, thermal stability, phase behavior, and chemical activity may be achieved by coordination-environment engineering and magnetic-ion diversification. Higher magnetic susceptibility does not, however, always translate into greater performance.^25^ For example, highly paramagnetic lanthanide compounds may show increased density or viscosity, and altering ligand design may enhance processability without significantly boosting magnetization. Therefore, rather than just increasing magnetic susceptibility, future MIL design should optimize magnetic response while accounting for viscosity, thermal and chemical stability, and cost.^23^

#### 2.1.3. Third generation: functionalization of cations *vs.* anions

The design approach for the third generation of MILs changed from merely altering the magnetic ion to selectively functionalizing the cation, anion, or both ionic components. This method allows for the independent tuning of hydrophobicity, polarity, viscosity, conductivity, solubility, interfacial activity, and molecular recognition while maintaining magnetic behavior. As a result, MILs can be created as task-specific materials where the functionalized ionic component dictates the intended use and the magnetic center provide field responsiveness.^26^

In order to provide application-specific groups without directly disrupting the magnetic anion, cation functionalization is especially helpful. While –NH_2_, –OH, –COOH, ether, and amide groups can offer particular interactions with metal ions, biomolecules, gasses, or organic contaminants, long alkyl chains, for instance, boost hydrophobicity and surface activity. Molecular functionalization can couple magnetic response with self-assembly and interfacial activity, as demonstrated by Kulshrestha *et al.*'s report of paramagnetic surface-active ILs in which modification of the cation/anion with long hydrocarbon chains promoted the formation of micelles, vesicles, and rod-like aggregates.^27^

Anion functionalization, on the other hand, offers direct control over the magnetic coordination environment. Ligand-field strength, spin state, magnetic moment, hydrophobicity, and thermal stability can all be changed by changing the ligands surrounding Fe^3+^, Co^2+^, Ni^2+^, or lanthanide centers. Recent Co^2+^/Ni^2+^ MILs with functionalized coordination environments, for instance, showed effective magnetic moments of roughly 2.78–5.16 µB, indicating that ligand architecture and metal identity both influence magnetic behavior. Ligand engineering can overcome the traditional trade-off between high magnetic responsiveness and poor fluidity, as demonstrated by functionalized diglycolic-acid ester MILs, which also obtained viscosities as low as 198.3 cP and thermal stability close to 345 °C.^16^

Additionally, functionalization can enhance the useful characteristics of MILs instead of just boosting magnetization, as recent research has shown. Compared to many previous hydrophilic MILs, hydrophobic MILs with functionalized diglycolate ligands have been produced with reduced viscosity, improved magnetic response, and heat stability, making them more appropriate for aqueous extraction and separation. As a result, the third-generation method proves that the location of the functional group introduction, cation, anion, or both, is just as crucial as the magnetic ion's identity.^11^

Thus, the cation–anion functionalization has changed MIL design from being mainly magnetism-centered toward the targeted modification of specific ionic components to tune individual physicochemical properties, such as hydrophobicity, viscosity, conductivity, solubility, and molecular recognition. The third generation is therefore defined by the location and chemical role of introduced functional groups, rather than by the simultaneous integration of multiple application-level functions. Nevertheless, more functionalities might increase the cost, molecular weight, viscosity, and synthesis procedures.^28^ Thus, the ideal MIL should balance magnetic responsiveness, viscosity, conductivity, thermal/chemical stability, selectivity, recyclability, toxicity, and synthetic complexity in addition to maximizing magnetic susceptibility. For the upcoming generation of MILs, this structure–property trade-off is a crucial path.^29^

Functionalization can improve the extraction selectivity, interfacial activity, and magnetic properties of MILs, but it may also increase environmental toxicity. For example, an ecotoxicological study of cholinium-based MILs reported 30 min EC_50_ values of 0.76–69.54 mg L^−1^ for the inhibition of *Vibrio fischeri* bioluminescence, depending on cation structure and metal-containing anion. The longer alkyl chain was associated with increased toxicity, whereas the MnCl_4_^2−^-based systems were generally less toxic than several other metal-containing analogues.^96^ These findings highlight that functionalization should be optimized not only for magnetic response and fluidity but also for environmental compatibility.

#### 2.1.4. Fourth generation: dual-paramagnetic MILs

By integrating two paramagnetic centers into the same ionic-liquid architecture, usually through magnetic cation–magnetic anion pairings, the fourth generation of MILs marks a substantial advancement from single-center systems. In addition to offering additional possibilities for adjusting hydrophobicity, viscosity, phase behavior, extraction selectivity, and thermal stability, this approach raises the density of magnetic centers and can significantly improve magnetic susceptibility. Dual-paramagnetic MILs, such as lanthanide-, Co^2+^, or Mn^2+^-based systems, can have the same magnetic ion in both ionic components, according to recent research, offering a more straightforward path to high magnetic responsiveness.^26^

Wu and Shen reported one of the most significant developments: they created dual-lanthanide MILs with the general formula [Ln(TODGA)_3_][Ln(hfac)_4_]_3_, where TODGA stands for *N*,*N*,*N*′,*N*′-tetra(*n*-octyl)diglycolamide and Ln = Tb, Dy, Ho, Er, Tm, and Yb. When the same lanthanide ion was incorporated into both the cationic and anionic components, the study reported an approximately fourfold enhancement in the specific magnetic parameter measured relative to similar lanthanide–halide MILs. This enhancement should not be interpreted as a fourfold increase in effective magnetic moment, magnetic susceptibility, and saturation magnetization simultaneously.^30^ However, this enhancement should be interpreted in terms of the specific magnetic parameter reported in the original study, rather than as a general fourfold increase in all magnetic properties. A rigorous quantitative comparison also requires consistent normalization (*e.g.*, on a molar or mass basis), measurement temperature, and applied magnetic field. Therefore, the reported enhancement is presented here as a system-specific finding, and direct quantitative comparisons should be made only when the relevant measurement conditions and normalization bases are equivalent.

Qiao *et al.* used the dual-Co^2+^ MIL [Co(DMBG)_2_][Co(hfac)_3_], in which Co^2+^ centers were integrated into both ionic components, to illustrate a complementary strategy. The material has a melting point of 341 K (68 °C) and a magnetic susceptibility of about 7.2 emu K mol^−1^. The MIL was hydrophobic, reddish in color, and easily recoverable in addition to having increased magnetism, proving that dual magnetic centers can offer both magnetic and application-specific activity.^31^ The dual-Co^2+^ MIL [Co(DMBG)_2_][Co(hfac)_3_] has a melting point of 341 K (68 °C), limiting its direct application in room-temperature liquid-phase extraction. Heating may be required to achieve fluidity, increasing energy consumption and operational complexity. However, it may still be useful in solid-phase applications, such as magnetic material development or as a precursor for functional magnetic materials, provided its stability and performance are verified. For room-temperature liquid-phase extraction, MILs that remain liquid at ambient temperature are more suitable.

The idea was expanded to include Mn(ii)-based dual-paramagnetic MILs, denoted as [Mn(C_*n*_im)_4_][Mn(hfac)_3_]_2_ (*n* = 2, 6, and 8). Notably, the C_3_ and C_6_ derivatives demonstrated strong hydrophobicity and remained liquid at normal temperature, which qualifies them for liquid–liquid microextraction. This work demonstrates a significant design benefit: the fluid nature needed for extraction procedures does not always have to be sacrificed in order to increase magnetic-center density.^32^

Dual-paramagnetic MILs can exhibit substantially enhanced magnetic response; for example, dual-lanthanide systems showed approximately fourfold higher magnetic response than comparable lanthanide-halide MILs, while the dual-Co^2+^ MIL [Co(DMBG)_2_][Co(hfac)_3_] exhibited a reported magnetic susceptibility of approximately 7.2 emu K mol^−1^ and a melting point of 341 K (68 °C).^30^ However, these gains must be balanced against fluidity and stability: the Co-based MIL melts above room temperature, whereas selected dual-Mn^2+^ derivatives remain liquid at room temperature.^32^ Quantitative viscosity and toxicity data under comparable conditions are needed to determine whether increased magnetic response translates into better overall performance.

As a result, fourth-generation MILs are distinguished here by the placement of paramagnetic centers in both ionic components, rather than by a claim that they necessarily follow all functionalized or multifunctional systems chronologically. The main benefit is the possibility of significantly increased magnetic response without the need for additional magnetic nanoparticles, up to almost four times in certain lanthanide systems. Future research, however, should concentrate on increasing magnetic susceptibility per unit viscosity and cost while preserving chemical stability, recyclability, room-temperature liquidity, and application-specific selectivity.^26^

#### 2.1.5. Fifth generation: multifunctional MILs

The fifth-generation category is defined by the deliberate integration of two or more complementary functional capabilities into a single MIL platform to achieve a specific application or synergistic performance that extends beyond functional-group modification alone. Unlike third-generation MILs, which are classified according to the functionalization of the cation, anion, or both, fifth-generation MILs are classified according to the combination of functional capabilities and their intended application. This category is not mutually exclusive from third-generation functionalization or fourth-generation dual-paramagnetic design, because functionalized ligands and multiple magnetic centers may also contribute to multifunctional performance.^26^

The ability of multifunctional MILs to combine magnetic separation and recognition into a single material is one of its main advantages. For instance, complementary-base recognition and magnetic manipulation were combined to create an oligonucleotide-functionalized hydrophobic MILs (OSMILs) for sequence-specific DNA extraction. During separation, the OSMILs produced particles that were around 897 nm in size, and an external magnet could gather them in about three seconds.^33^

Combining optical and magnetic features can potentially result in multifunctionality. Because Ln^3+^ ions can concurrently produce paramagnetism and distinctive luminescence, lanthanide-containing MILs are very appealing. For instance, at 352 nm excitation, the Tb-containing system [Tb(TODGA)_3_][Tb(hfa)_4_]_3_ displays green emission, with a significant contribution from the anionic coordination environment. These devices show that optical signalling and magnetic responsiveness can be provided by the same ionic architecture, creating opportunities for magnetically controlled optical sensing and imaging.^34^

Additionally, magnetic extraction along with analytical detection has been added to the multifunctional concept. In 2024, inorganic and organic mercury species in water and wine were simultaneously separated and determined using a MIL-based effervescence-assisted liquid–liquid microextraction method that used [P_66614_][FeCl_4_]. Magnetic responsiveness can simplify analytical procedures while preserving selective extraction; just 75 µL of MIL was needed, and the extracted phase could be quickly separated magnetically without centrifugation.^35^

Similarly, the selective detection of Cd and Zn chlorocomplexes in saline fluids has recently been accomplished *via* dispersive liquid–liquid microextraction based on MIL. Centrifugation was not necessary because to the use of methyltrioctylammonium tetrachloroferrate, [N_1,8,8,8_][FeCl_4_], which offered both extraction functionality and quick magnetic phase recovery. This demonstrates a significant practical benefit of multifunctional MILs: the ability to combine physical separation and chemical selectivity into a single extraction platform.^36^

Additionally, multifunctionality is moving beyond sensing and extraction to include biological and theranostic uses. A recent MIL-carbon nanohorn nanocomplex used carbon nanohorns, indocyanine green, and [Bmim][FeCl_4_] to combine magnetic, fluorescent, photothermal, and anticancer properties. In addition to enabling magnetic guiding, near-infrared fluorescence, and photothermal treatment, the device demonstrated a photothermal conversion efficiency of 63% and stayed dispersed in water for about seven days. These findings demonstrate how MIL-based platforms can incorporate several external stimuli and therapeutic activities within a single material design.^37^

Multifunctionality does not, however, always result in better overall performance. While excessive functionalization may decrease ionic mobility, the addition of functional groups, metal centers, or secondary components can increase molecular weight, viscosity, synthesis difficulty, expense, and possible toxicity.^38^ Therefore, the synergistic integration of complementing functions without sacrificing transport and stability is the proper design requirement rather than the largest number of functions. Therefore, the field should shift toward application-specific MILs that simultaneously maximize magnetic response, selectivity, optical/electrochemical activity, viscosity, recyclability, and environmental compatibility.^39^

#### 2.1.6. Emerging generation: metal-free and predictive magnetic ionic liquids

Through two complimentary approaches, metal-free organic-radical MILs and predictive computational design, the next generation of MILs is progressing beyond traditional metal-centered structures. Instead of transition-metal or lanthanide centers, stable organic radicals with unpaired electrons are the source of magnetic behavior in metal-free MILs, which lessens reliance on potentially hazardous or costly metals. In parallel, density functional theory (DFT) and molecular dynamics (MD) simulations have been applied to investigate the structures, interactions, transport properties, and application-specific behavior of experimentally studied MILs. Machine learning (ML), although promising for predicting ionic-liquid properties and accelerating molecular design, has been demonstrated predominantly for conventional ionic liquids rather than systematically validated MIL datasets. Accordingly, this section distinguishes experimentally demonstrated advances in MILs from computational approaches that remain prospective for MIL-specific prediction and inverse design (Fig. 2).^26^

**Fig. 2:**
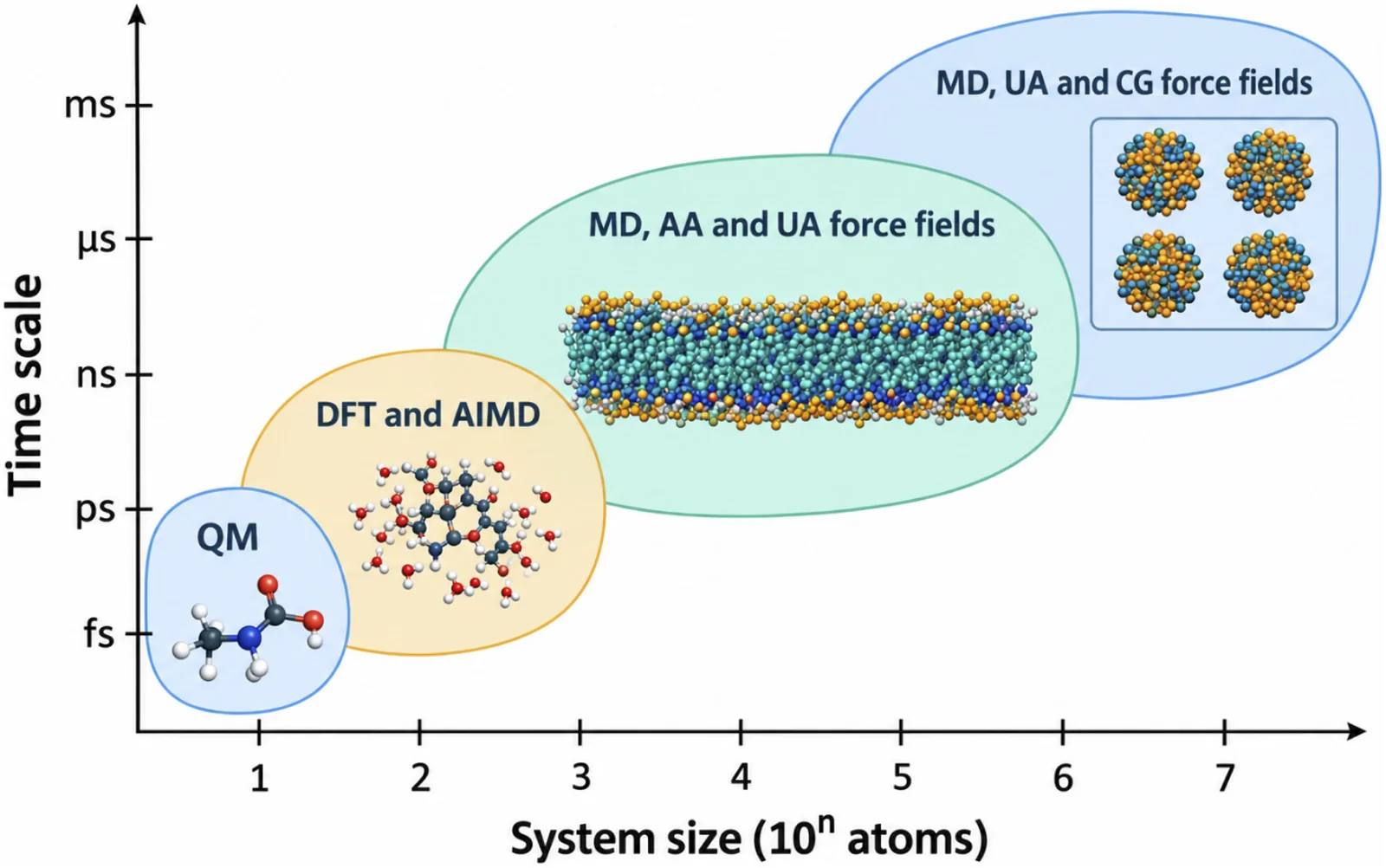
Modelling of IL-based systems: choice of suitable computational methods depending on the sizes of the systems of interest and time scales, reproduced from ref. 26 with permission from the American Chemical Society, *Chem. Rev.*, 2024, **124**(6), 3392–3415, copyright 2024.

Yoshida *et al.* first used the stable TEMPO-derived radical anion, TEMPO-OSO_3_^−^, in conjunction with alkylimidazolium cations such as [C_2_C_1_im]^+^, [C_4_C_1_im]^+^, [C_2_C_1_im]^+^, and [C_2_C_1_im]^+^ to illustrate the idea of metal-free MILs. Without a metal ion, the radical center's S = 1/2 allows for magnetic responsiveness. It is interesting to note that while the C_2_ analog crystallized at a melting point of roughly 330.15 K (57 °C), the C_4_, C_6_, and C_8_ derivatives were liquids at ambient temperature. These systems showed that organic molecule design is the only way to accomplish magnetic responsiveness and fast magnetic phase separation.^40^

Chiral MILs, which are characterized by their simultaneous chiral and magnetic properties, have recently been used to pioneer a novel aqueous two-phase system. This novel strategy uses a set of MILs based on amino acids, namely [C_n_C_4_im-TEMPO]-[l-Pro] (where *n* = 2, 3, and 4), a special combination created by Yao *et al.* In this work, an efficient aqueous biphasic system designed for enantiomeric separation was created by combining the MILs with inorganic salts. A technique that produced quantifiable amounts in grams was achieved through the optimization of extractive resolution conditions.^41^

Temperature-responsive metal-free MILs based on polypropylene glycol (PPG_1000_) form another significant class. Tetracycline antibiotic enrichment and trace analysis in cow's milk have been conducted using these compounds. Their magnetic responsiveness makes extraction phase easy, and their temperature-dependent hydrophilicity allows for regulated phase separation and recovery.^42^

Optimizing MIL structures, analyzing ion-pairing configurations, assessing electronic characteristics, and locating favorable interaction sites are the core applications of DFT simulations. For instance, DFT optimization and vibrational calculations were used in recent computational work on [C_3_mim]FeCl_4_ to determine a stable molecular configuration of the MIL and connect its structure to behavior found in experiments.^43^ Similarly, González-Veloso *et al.* used DFT, SAPT, and DLPNO-CCSD(T)-based local energy decomposition to study [Bmim][FeCl_4_], [Bmim][FeBr_4_], [Emim][FeCl_4_], [ZnCl_3_]-, and [SnCl_3_]-containing systems. In most systems, electrostatic interactions were shown to be the main contribution, followed by dispersion interactions. The interaction-energy trends were further validated by comparison with higher-level DLPNO-CCSD(T) simulations. The potential of energy decomposition for determining structure–property links in MILs was demonstrated by the significant correlation between estimated interaction energies and experimentally measured melting points.^44^

Molecular dynamics simulations are a crucial addition to QM calculations for bigger systems and dynamic features. Density, diffusion, viscosity, ion aggregation, radial distribution functions, transport characteristics, and gas–MIL interactions can all be assessed by studying thousands of atoms over significantly longer length and time scales using classical MD. The development and validation of fixed-charge force-field parameters for phosphonium-based MILs, such as [P_66614_][FeCl_4_], [P_66614_][FeBr_4_], [P_66614_]_2_[MnCl_4_] and [P_66614_]_3_[GdCl_6_], was a significant recent development that was reported in 2025. Multivalent paramagnetic anions significantly affect gas solvation and mobility, according to MD simulations; phosphonium MILs demonstrated favorable solvation behaviors toward CO_2_ and SO_2_.^45^

The use of MIL-based computer modelling has also been extended in recent simulations beyond intrinsic liquid properties to interfacial and separation processes. In 2025, a magnetic ionic-liquid microemulsion collector based on [C_12_mim]FeCl_4_ was designed using coarse-grained MD and tests, helping to comprehend the structure of the MIL-containing microemulsion and supporting its formulation for coal flotation.^46^ Similarly, a 2024 study that combined experimentation and molecular simulation revealed that Fe-containing MILs can alter hydrogen-bond networks and improve coal's swelling behavior, proving the value of molecular modelling for comprehending MIL-substrate interactions.^47^

Additionally, the use of MD simulations to comprehend the biomolecular interactions of MILs is growing. Molecular docking and long-timescale MD simulations were used in a 2024 Chemical Science study to examine choline-based MIL interactions with calf-thymus DNA. While experimental measurements confirmed the observed compaction of DNA, the simulations, which extended to 1 µs, offered molecular-level information on MIL–DNA binding and structural alterations.^48^

Accurately treating paramagnetic centers, particularly their open-shell electronic structures and polarization effects, continues to be a serious issue. However, prediction of MIL structure, transport, gas capture, interfacial behavior, and biomolecular interactions is becoming more and more possible thanks to the creation of verified force fields and the integration of DFT with MD. The 2026 MD study on greenhouse-gas capture is especially important since it shows that computational models are no longer limited to simple MIL ion pairs and can now handle multivalent magnetic anions and application-relevant gas selectivity.^45^

DFT was utilized to improve the molecular structure and explore the electronic properties of the MIL in experimental-computational investigation for [C_3_mim]FeCl_4_. Interpretation of the experimentally synthesized material and its interaction with coal during swelling and pyrolysis was supplemented by the molecular-level information supplied by the computed optimal structure.^43^ A related investigation on [C_12_mim]FeCl_4_ used magnetic-field-assisted salting-out to facilitate MIL recovery, while simulation and differential scanning calorimetry were used to identify the FeCl_4_^−^ structure and suitable synthesis ratio.^49^

Predictive modelling of ILs, more generally, offers a pathway that can eventually be applied specifically to MILs. A 2024 deep-learning study analyzed 303 880 potential cation–anion pairings using 73 847 experimental data points covering 2917 ILs from 1213 references. The density, viscosity, surface tension, and melting temperature values obtained by the optimized ensemble neural-network model were 0.993, 0.907, 0.931, and 0.875, respectively. This dataset shows that multi-property inverse design is feasible and offers a methodological basis for future MIL-specific datasets, even if it was not limited to MILs.^50^

It is important to distinguish the demonstrated applications of computational methods in MIL research from the broader predictive capabilities reported for conventional ionic liquids. DFT and MD have already been applied to specific MIL systems to investigate molecular structure, ion interactions, transport behavior, gas solvation, and interfacial phenomena, in some cases alongside experimental validation. In contrast, the deep-learning model discussed above was developed using a broad conventional ionic-liquid dataset and should not be interpreted as direct experimental validation of ML predictions for MILs. The extension of such models to MILs requires representative datasets incorporating magnetic susceptibility, viscosity, phase-transition temperatures, and other relevant properties, together with appropriate treatment of paramagnetic centers and independent experimental validation. Thus, ML currently represents a promising prospective tool for MIL design rather than a universally established predictive framework for these materials.^50^

Thus, metal-free and predictive MILs mark a significant transition from empirical synthesis to data-driven and molecularly informed design. While computational methods can lower the number of experimental candidates needed, metal-free radical MILs can lessen dependency on transition and rare-earth metals.^51^ Radical stability, oxidation resistance, magnetic-field response, restricted MIL-specific datasets, force-field transferability, experimental validation, and uncertainty quantification are among the significant obstacles that still need to be overcome. Therefore, rather than depending just on computational predictions, the next generation should concentrate on DFT/MD-guided and ML-assisted inverse design, followed by experimental validation of magnetic susceptibility, viscosity, conductivity, thermal stability, toxicity, and recyclability.^9^

The generational evolution of MILs is summarized in Table 1, which focuses on how their design tactics, magnetic properties, physicochemical qualities, and functional applications have changed over time. Thus, the emerging generation of MILs encompasses experimentally demonstrated metal-free radical systems and computationally guided strategies for developing next-generation magnetic liquids. While DFT and MD have already contributed to understanding specific MIL structures and behaviors, the application of ML to MIL-specific property prediction remains at an early stage. Future research should focus on reliable predictive models and validation of key physicochemical properties.

**Table 1:** MILs generations: design evolution, key properties, and values

Generation	Metal/design strategy	Representative system	Magnetic properties & values	Physicochemical values	Insight/trade-off
1st gen	Single magnetic ion [FeCl_4_]^−^	8 MILs, various cations	*χ*_MT_ 3.49–4.50 emu mol^−1^ K Oe^−1^; *µ*_eff_ ∼ 5.66 µB	*D* ∼ 7.55 × 10^−1^ cm^2^ s^−1^; ket 3.60 × 10^−3^ cm s^−1^	Established metal-ion incorporation concept; balance Fe^3+^ with transport properties^19^
2nd gen	Coordination environment & metal diversity (Co^2+^, Ni^2+^, Mn^2+^, Fe^3+^, Gd^3+^, Dy^3+^, Ho^3+^)	30 MILs (diglycolamide ligands)	*µ*_eff_ 4.71–21.08 µB; Dy/Ho > Fe-based systems	Thermal stability: 192–235 °C depending on metal	Co/Ni NTf_2_-based MILs: *µ*_eff_ 2.78–5.16 µB; changing metal > changing ligand for spin state^21^
3rd gen	Cation/anion functionalization (–NH_2_, –COOH, –OH, alkyl chains)	Co^2+^/Ni^2+^ NTf_2_ + functionalized ligands	*µ*_eff_ 2.78–5.16 µB	Viscosity as low as 198.3 cP at 22.8 °C; stability up to 345 °C	Task-specific MILs; functionalized diglycolate overcomes viscosity–magnetism trade-off^21^
4th gen	Dual-paramagnetic (magnetic cation + magnetic anion)	[Ln(TODGA)_3_][Ln(hfac)_4_]_3_; [Co(DMBG)_2_][Co(hfac)_3_]	∼4× improvement *vs.* lanthanide-halide MILs; [Co] *χ* ∼ 7.2 emu K mol^−1^	Mn dual-MILs: hydrophobic, liquid at RT; mp ∼ 341 K for [Co] dual	Multiply paramagnetic centers without extra nanoparticles; cooperative design^30^
5th gen	Multifunctional (magnetic + catalytic/optical/sensing/bio)	OSMIL, Tb-TODGA, [P_66614_][FeCl_4_] MIL-based platforms	OSMIL particle size ∼ 897 nm; magnetic phase in ∼3 s; Tb emission at 352 nm excitation	75 µL MIL used for Hg extraction; photothermal efficiency 63%; stable in water ∼7 days	Combines extraction, detection, optical, and therapeutic functions into one platform^35^
Emerging gen	Metal-free organic radical + ML/DFT-guided design	TEMPO-OSO_3_^−^ + [C_*n*_C_1_im]^+^; PPG_1000_-based; amino acid MILs	S = 1/2 radical; C_4_/C_6_/C_8_ liquid at RT; C_2_ mp ∼ 330 K	DFT *R*^2^: density 0.993, viscosity 0.907; MD simulations up to 1 µs	Reduces reliance on metals; MIL predicts 303 880+ ion pairs; data-driven design^40^

### 2.2. Route-based synthesis of magnetic ionic liquids

Recent studies on MILs have advanced from traditional manufacture by straightforward metal-halide inclusion to quick, solvent-minimized, immobilized, bimetallic, and task-specific synthesis methods. The creation of the magnetic ionic phase, as well as its viscosity, phase stability, magnetic responsiveness, accessibility of functional sites, and recyclability, are increasingly determined by the synthetic pathway. Therefore, recent research shows a shift from preparing MILs mainly for their magnetic response to creating MIL-based hybrid materials and application-specific magnetic liquids.^52^

#### 2.2.1. Mechanochemical or solvent-free synthesis

As compared to traditional solution or metathesis methods, direct ball-milling synthesis of molecular MILs is still far less established, and a review should make this discrepancy evident. A recent work showed a phosphonium-based MIL as curing agent for graphene nanoplatelet/carbon-nanotube epoxy nanocomposites, which were made using solvent-free ball milling. When compared to the similar GNP-alone system, electrical conductivity rose by about six orders of magnitude when only 3 phr of GNP/CNT filler was added. At a thickness of 1.5 mm, the MIL-cured composite with GNP/CNT at 2.5/0.5 phr showed an effective absorption bandwidth of 5.18 GHz and a minimum reflection loss of −23.61 dB.^53^

A one-pot, solvent-free heat/stir synthesis of twelve transition-metal MILs based on Co^2+^/Ni^2+^–[NTf_2_^−^] salts and N/O-donor ligands was shown by Abbasi *et al.* (2024), as shown in Fig. 3. Most methods formed homogenous products in around 12 hours after the MILs were prepared at less than 50 °C. They were then vacuum-dried for 24 hours. The produced MILs showed thermal stability up to 345 °C, viscosities as low as 198.3 cP at 22.8 °C, and effective magnetic moments of 2.78–5.16 µB. Interestingly, certain MILs could also be produced *in situ* in water in less than 30 seconds, proving that MIL production can happen quickly without the need for traditional organic reaction media or anion-exchange purification procedures.^21^

**Fig. 3:**
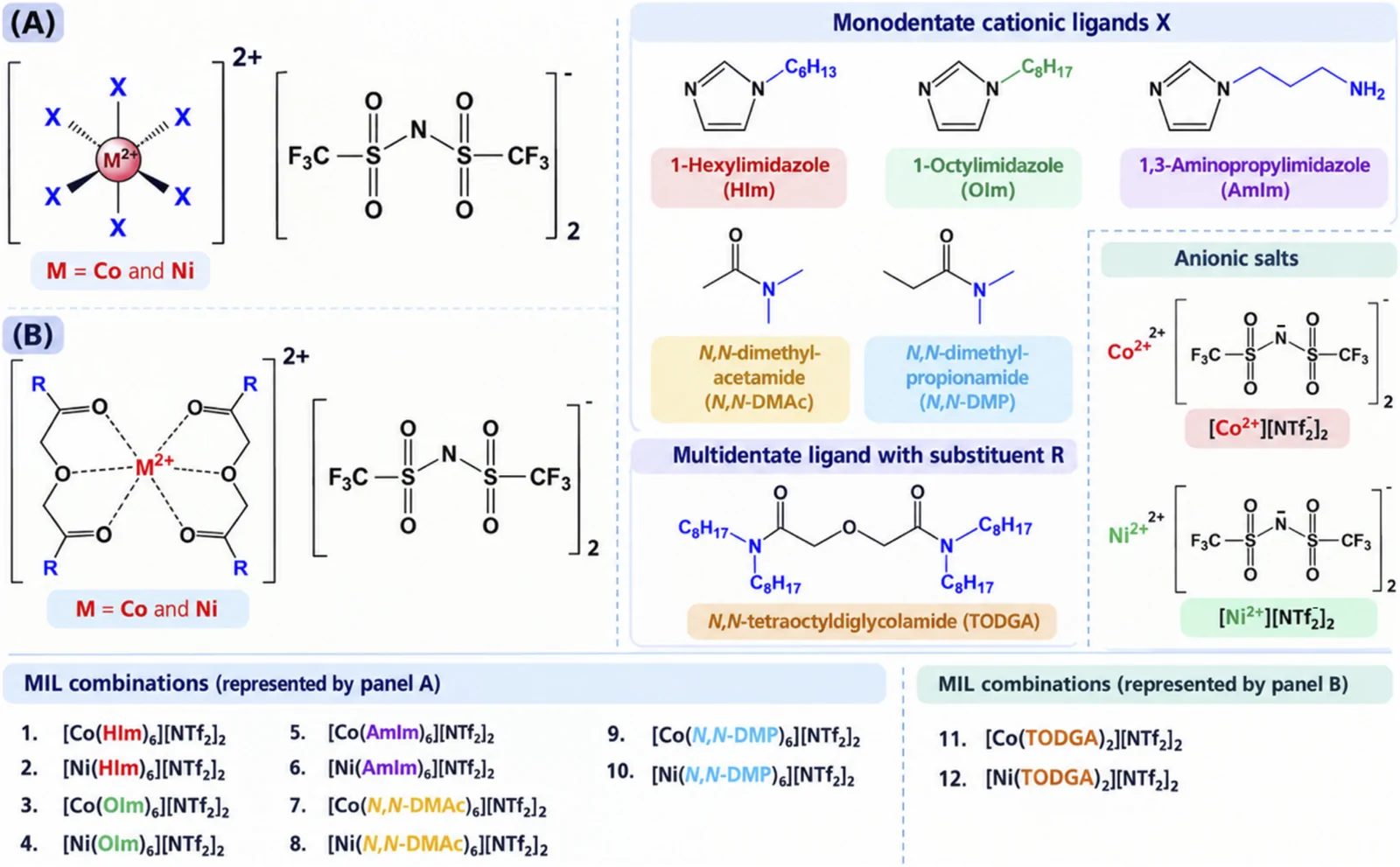
Chemical structures and numbering scheme of MILs based on (A) monodentate cationic ligands and (B) diglycolamide ligands, reproduced from ref. 21 with permission from the Centre National de la Recherche Scientifique (CNRS) and the Royal Society of Chemistry, *New J. Chem.*, 2024, **48**(15), 6571–6581, copyright 2024.

Knoop *et al.* showed how to prepare [C_4_C_1_Im][FeCl_4_] neatly by solvent-free reflux, with the MIL production taking around 48 hours. This shows that removing the reaction solvent is possible, although it may still require lengthy processing periods.^20^ On the other hand, Gu *et al.* described a simple two-step synthesis of an imidazolium/[FeCl_4_]^−^ MIL with an electrochemical window of about 5.0 V and a paramagnetic susceptibility of 1.82 × 10^−1^ emu g^−1^, proving that simpler preparation can produce functionally stable MILs.^54^ These experiments show that solvent elimination is technically possible, but since MILs are still mostly synthesized by heat mixing, stirring, or reflux rather than direct mechanical activation, solvent-free synthesis should not be confused with mechanochemical synthesis. In order to establish reproducibility and scalability as a true MIL-synthesis technology, future mechanochemical systems should rigorously link milling energy, milling time, temperature, product homogeneity, yield, and phase stability.^23^

Despite its advantages, direct ball-milling synthesis of neat molecular MILs remains underdeveloped, with limited reproducibility and comparability across studies. Reaction outcomes depend on milling conditions, energy transfer, and temperature, while incomplete conversion, agglomeration, media contamination, and undesired phase formation may affect product quality.^20^ To improve reproducibility, future studies should standardize and explicitly report several key process parameters: (i) the milling equipment and operating mode, including mill type, frequency or rotational speed, and milling duration; (ii) the milling vessel and ball materials, ball diameter, number of balls, and ball-to-powder mass ratio; (iii) precursor identity, purity, stoichiometric ratio, initial particle size, and total batch mass; (iv) the use and amount of any liquid-assisted grinding agent, if applicable, to distinguish neat mechanochemistry from liquid-assisted grinding; and (v) temperature evolution, atmosphere, milling interruptions, and cooling intervals. Product yield, phase purity, structural identity, residual precursors, batch-to-batch variation, and energy consumption should also be evaluated using appropriate characterization techniques. Systematic parameter optimization, replicate experiments, and comparisons with conventional synthesis routes are essential to establish structure–property relationships, assess sustainability, and enable scalable mechanochemical production of MILs.

Although solvent-free and one-pot routes can reduce organic solvent consumption, their overall sustainability remains dependent on product yield, energy demand, purification requirements, and precursor cost. For example, the reported one-pot synthesis of Co^2+^/Ni^2+^–[NTf_2_^−^]-based MILs required approximately 12 h of reaction time below 50 °C, followed by 24 h of vacuum drying, whereas certain aqueous *in situ* preparations were completed within 30 s. These differences highlight the potential for reducing processing time and solvent use.

#### 2.2.2. Ionic-liquid-based magnetic coordination polymers and MOF-type materials

Coordination frameworks, which combine the adjustable ionic environment with the porosity and structural stability of coordination polymers, are increasingly being employed to immobilize or confine ionic-liquid functionalities beyond discrete magnetic ionic liquids.^55^ Importantly, ionic-liquid-functionalized MOFs (IL-MOFs) should be distinguished from genuine MILs. Genuine MILs are liquid salts whose magnetic response originates from paramagnetic ions or stable radical species, whereas IL-MOFs are hybrid materials in which ionic-liquid components are incorporated into a porous solid framework. Therefore, IL functionalization alone does not qualify a MOF as a MIL, even when the framework contains magnetic metal centers. These materials are included in this review as related hybrid systems because they demonstrate complementary strategies for controlling ionic environments, magnetic functionality, porosity, and separation or catalytic performance. By substituting a sulfonate/imidazolium-containing IL for coordinated formate groups, Jia *et al.* (2023) functionalized MOF-808, resulting in a proton conductivity of 1.28 × 10^−1^ S cm^−1^ at 60 °C and 100% RH, indicating effective proton transport through IL-induced hydrogen-bond networks.^56^ Through Cr^3+^–N coordination, Zou *et al.* (2021) anchored a Zn-containing imidazolium bromide IL onto MIL-101(Cr), resulting in a surface area of 1146 m^2^ g^−1^, 97.3% propylene-carbonate production, and 100% selectivity for CO_2_/epoxide cycloaddition, with activity maintained over five cycles.^57^ These examples represent IL-functionalized porous frameworks rather than genuine MILs, because their reported functions arise primarily from framework porosity, ionic functionality, and catalytic activity rather than from a demonstrated liquid-state magnetic response.

Using a polyoxometalate bridge, Chen *et al.* (2022) added a SO_3_H-functionalized IL to MIL-101(Cr), which prevented active-component leaching and produced combination Lewis/Brønsted acidity for the generation of biodiesel.^58^ In order to show that IL incorporation can alter water-transfer behavior and allow the MOF to operate in heat-to-work water-transfer processes, Han and Chakraborty (2024) added ILs to HKUST-1 while maintaining the framework structure.^59^ In order to create cooperative Zn Lewis-acid/Br^−^ Lewis-base sites, Xiang *et al.* (2021) grafted [AEMIM]Br onto dual-linker ZIF-8. The optimized IL-ZIF-8 achieved 97% cyclic-carbonate yield, although DFT calculations revealed an epoxide ring-opening barrier of just 11.5 kcal mol^−1^.^60^ These studies further illustrate the value of IL-MOF composites for catalysis, proton transport, and water management; however, their reported performance should not be interpreted as evidence of MIL-specific magnetic behavior.

A family of hydrophobic transition-metal-chelate MILs based on Co^2+^ and Ni^2+^ complexes with [NTf_2_]^−^ counterions, such as [Co(HIm)_6_][NTf_2_]_2_, [Co(OIm)_6_][NTf_2_]_2_, [Co(AmIm)_6_][NTf_2_]_2_, [Co(DMAc)_6_][NTf_2_]_2_ and [Co(DMP)_6_][NTf_2_]_2_ were described recently. These systems had effective magnetic moments of roughly 2.78–5.16 µB and were made for quick formation.^21^ Unlike the preceding IL-MOF composites, these systems are relevant to MIL research through their molecular magnetic centers; their classification as MILs depends on confirmation of their liquid state under the relevant operating conditions.

Using a ship-in-bottle method, Jiang *et al.* (2021) created a dicationic imidazolium IL directly within MIL-101; the composite had a BET surface area of 1162 m^2^ g^−1^, CO_2_ uptake of 47.4 cm^3^ g^−1^ at 273 K, and 92.5% propylene-carbonate production at 100 °C and 1.0 mPa.^61^ While maintaining the MIL-100(Fe) structure, Mortazavi *et al.* (2021) contained a multi-SO_3_H-functionalized IL within MIL-100(Fe), producing 0.9 mmol g^−1^ acid sites and achieving 99% styrene-oxide conversion in 3 hours under solvent-free circumstances.^62^ Although these frameworks contain Fe or other metal centers, the presence of such centers alone does not establish that the resulting composites are magnetic ionic liquids; their principal relevance here is the functional integration of ILs with porous coordination materials.

To demonstrate the synergistic contribution of the UiO-66 framework and IL domains, Yin *et al.* (2023) created UiO-66-based porous ionic liquids in which the permanent MOF porosity was preserved after IL functionalization. The final material combined molecular extraction with catalytic oxidative desulfurization.^63^ Zhang *et al.* (2024) modified the local electronic environment of Fe-porphyrin sites by incorporating a carboxyl-functionalized imidazolium IL into Fe-porphyrinic Zr-MOF-526. IL_200_@MOF-526 produced 14.0 mmol g^−1^ CO in 72 hours with 97% selectivity under visible light without the use of an external photosensitizer.^64^ These examples are included because they demonstrate how IL-framework interactions can modify catalytic and separation performance, rather than because they necessarily exhibit the defining liquid-state magnetic properties of MILs.

In contrast to traditional MOF structures, Zhu (2025) reported IL-assisted one-step synthesis of mesoporous MOFs, demonstrating that suitably designed ionic liquids can acts as reaction media and structure-directing/coordination-modulating components, producing significantly enlarged mesoporous environments.^52^ Together, these studies show that coordination anchoring, covalent grafting, ship-in-bottle confinement, and *in situ* assembly can be used to incorporate IL into coordination polymers and MOFs, allowing for simultaneous control over porosity, magnetic/ionic character, catalytic sites, molecular recognition, and transport properties. Their inclusion broadens the discussion to related materials-engineering strategies while maintaining a clear distinction between porous IL-containing composites and genuine MILs.

Despite their promising catalytic performance, IL-functionalized MOFs require quantitative evaluation of synthesis yield, solvent consumption, energy demand, and production cost to establish their practical sustainability. Reported results include 97.3% propylene-carbonate production for IL-functionalized MIL-101(Cr), 99% styrene-oxide conversion using IL@MIL-100(Fe), and 97% cyclic-carbonate yield with IL-ZIF-8; however, these performance metrics do not independently establish environmental benefits. Accordingly, these systems are discussed as complementary IL-based hybrid materials, whereas claims concerning MIL-specific magnetic performance should be reserved for materials whose magnetic response and liquid-state behavior have been experimentally established.

#### 2.2.3. Sol–gel-assisted synthesis

By incorporating ionic-liquid functionality into silica or magnetic oxide networks, sol–gel processing has become a successful method for transforming mobile ionic-liquid phases into magnetically recoverable heterogeneous materials.^20^ The method combines the high surface area, structural stiffness, and magnetic separability of inorganic supports with the chemical tunability of ionic liquids.^16^ Early research showed that acidic imidazolium ionic liquids may be immobilized on cobalt-ferrite-containing mesoporous silica made by a reverse-microemulsion sol–gel method, resulting in magnetically responsive esterification catalysts.^65^ These products should be described as magnetic ionic-liquid-supported composites or immobilized ionic-liquid networks, rather than as molecular MILs, because the ionic-liquid functionality is anchored to a solid magnetic support and the resulting material is heterogeneous rather than a freely flowing magnetic salt.

A magnetic hybrid sol–gel ionic network nanoparticle (MINN) based on immobilized imidazolium poly linkers and silica-coated iron oxide nanoparticles was published in 2025. A magnetic ionic network containing sulfonic acid was produced by subsequent counter-anion exchange. For solvent-free esterification at 85 °C, the resultant catalyst may be utilized at just 0.1 mol%, producing 78–99% ester yields and being reusable for ten cycles without significantly losing its catalytic activity or magnetic susceptibility.^66^

More recently, ionic-liquid structures supported by magnetic silica have been investigated for catalysis and adsorption. For instance, recyclable magnetic nanocatalysts with a large number of accessible ionic sites were produced by immobilizing imidazolium-based dicationic ILs on Fe_3_O_4_@SiO_2_@dendritic mesoporous silica nanospheres.^67^

Farzam *et al.* synthesized an imidazolium IL immobilized on Fe_3_O_4_@MCM-41 using a one-pot sol–gel technique, demonstrating the strategy's environmental application. The final product had a high surface area of 1177.4 m^2^ g^−1^, a pore size of about 4.2 nm, a pore volume of 1.80 cm^3^ g^−1^, and a magnetic saturation of 31.5 emu g^−1^. Its use in magnetic micro-solid-phase extraction demonstrated the benefit of integrating mesoporosity, ionic-liquid functionality, and magnetic separation, with pesticide recoveries of 82.4–109.8% with detection limits of 0.04–1.63 ng L^−1^.^65^

Additionally, the idea has been expanded to include polymeric ionic-liquid networks in addition to molecular ionic liquids. To create magnetically sensitive catalysts with strong esterification activity and recyclability, Xue *et al.* polymerized poly(ionic liquids) directly on Fe_3_O_4_@SiO_2_ nanoparticles.^68^ More recently, Faraji *et al.* created a cross-linked magnetic ionic-network nanoparticle made of immobilized imidazolium-based poly(ionic-liquid) linkers and silica-coated iron oxide cores. Following sulfonic-acid functionalization, the catalyst maintained its catalytic activity and magnetic susceptibility for ten reuse cycles while operating at only 0.1 mol% and 85 °C in solvent-free conditions, yielding 78–99% ester.^66^

All of these investigations show that sol–gel-assisted immobilization offers a flexible way to combine mechanical stability, mesoporosity, magnetic recoverability, and ionic activity into a single heterogeneous design. The translational mobility characteristic of free MILs is inevitably reduced by immobilization, but the resulting materials are especially appealing when maintaining a fully liquid magnetic phase is not as important as rapid separation, low catalyst loss, structural durability, and repeated reuse.^18^

Although sol–gel-assisted synthesis offers promising catalytic performance and reusability of catalyst synthesis. Farzam *et al.* reported a surface area of 1177.4 m^2^ g^−1^ and saturation magnetization of 31.5 emu g^−1^, with pesticide recoveries of 82.4–109.8%, whereas Faraji *et al.* achieved 78–99% ester yields and retained catalytic activity over ten cycles under solvent-free reaction conditions.

#### 2.2.4. Microwave-assisted synthesis

Since the high ionic conductivity and dielectric responsiveness of ILs can enable effective microwave energy absorption, microwave irradiation offers quick volumetric heating and is especially appealing for ionic-liquid-based magnetic materials.^27^ However, microwave-assisted synthesis of real molecular MILs is still largely unexplored in comparison to traditional solvothermal, sol–gel, and precipitation methods; the majority of current research has been on IL-functionalized magnetic nanoparticles and hybrid magnetic materials.^20^ Accordingly, the nanoparticle-based examples discussed below demonstrate microwave-assisted preparation or functionalization of magnetic composites, not necessarily the synthesis of molecular MILs. The term “MIL” should be reserved for products whose ionic composition and liquid-state magnetic response are established; otherwise, use “IL-functionalized magnetic nanoparticles” or “magnetic IL-based composite.”

A significant development was reported in 2024 by Cagli *et al.*, who used a series of imidazolium-based ILs comprising chloride, [NTf_2_]^−^, and pyrrolide anions as reaction media to create a single-step microwave-assisted synthesis of IL-functionalized Fe_3_O_4_ nanoparticles. At 80 °C, the synthesis was carried out quickly, allowing for both *in situ* surface functionalization and magnetite production. With saturation magnetizations of 81 emu g^−1^ at 10 K and 73 emu g^−1^ at 300 K, the resultant nanoparticles were mostly Fe_3_O_4_ and showed superparamagnetic behavior. Surface chemistry, particle size, magnetism, and colloidal stability were all significantly impacted by the IL structure, yet the functionalized nanoparticles stayed evenly distributed in water and DMSO for around three months.^69^

Microwave irradiation was used in a recent 2024 study on hydrophobic IL-modified FeO nanoparticles to create magnetic IL-based adsorbents for the removal of heavy metals and dyes (Fig. 4). The final materials showed the viability of combining microwave processing with hydrophobic ionic-liquid functionalization of magnetic oxide particles.

**Fig. 4:**
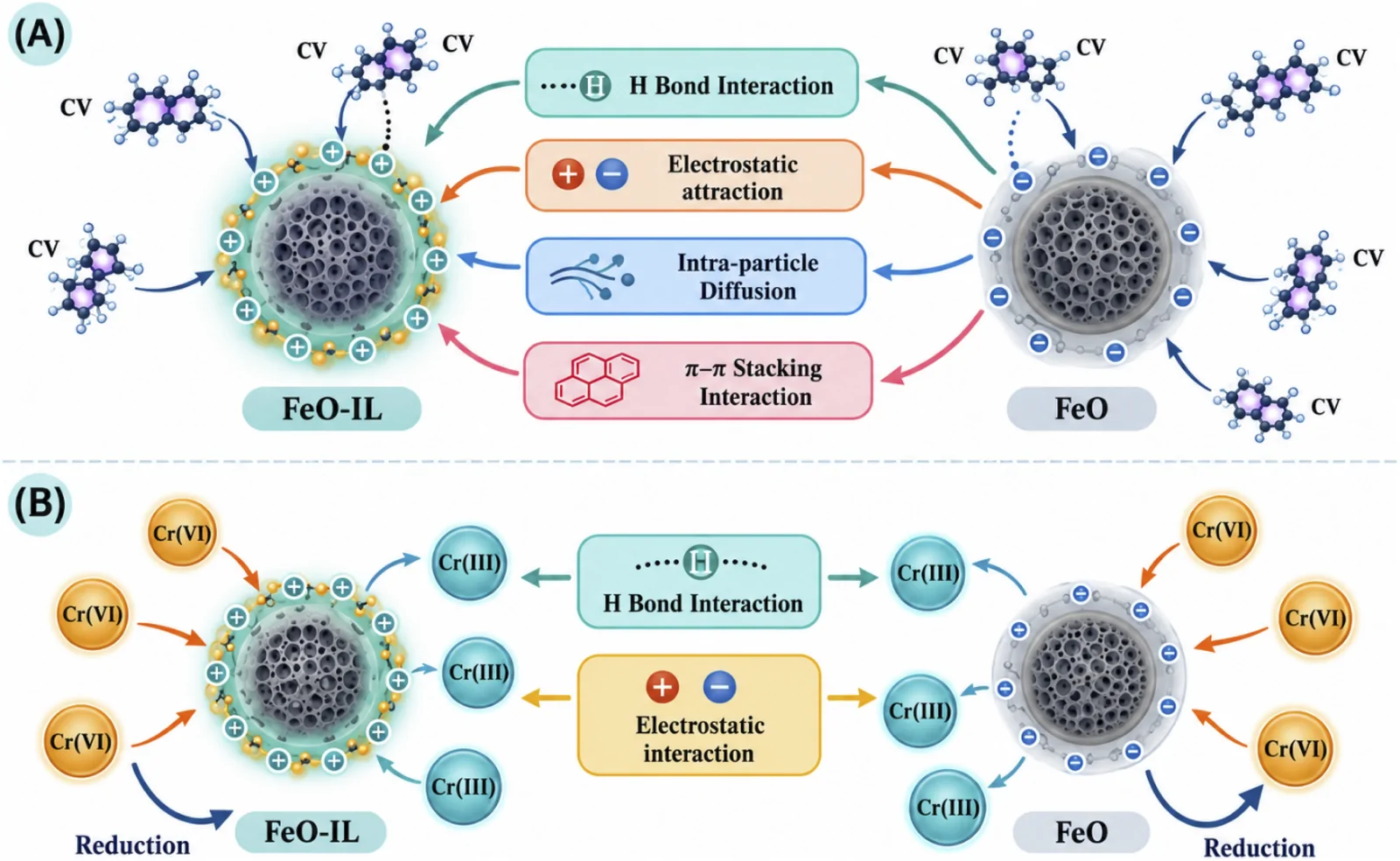
Mechanism of magnetic IL-based adsorbents for the removal of, (A) dye and (B) heavy metals, reproduced from ref. 70 with permission from the Royal Society of Chemistry, *Environ. Sci.: Adv.*, 2024, **3**(3), 480–501, copyright 2024.

Additionally, more intricate IL-containing magnetic constructions have been combined with microwave irradiation. IL-coated Fe_3_O_4_/multi-walled carbon nanotubes were used in microwave-assisted multicomponent synthesis in 2024 as magnetically recoverable catalysts. The CNT–Fe_3_O_4_–IL architecture demonstrated that microwave irradiation may be paired with magnetic IL-containing materials for fast heterogeneous synthesis, offering the combined benefits of ionic-liquid-mediated catalysis and magnetic separation.^71^

[BIM][FeCl_4_] and [OIM][FeCl_4_] were recently studied under 2.45 GHz microwave irradiation. 0.1 weight percent aqueous dispersions achieved roughly 81 and 93 °C, respectively, at 400 W for 60 seconds. The final temperature rose by about 10–15 °C when the MIL concentration was increased from 0.05 to 0.10 weight percent. Rather than magnetic losses, ionic conductivity and dipolar polarization were the main causes of the reaction. This new finding implies that MIL synthesis can be developed for energy-transfer and magnetically responsive heating applications in addition to magnetic separation.^72^

Hou *et al.*, who showed that [Bmim][BF_4_] may serve as a stabilizing medium and microwave absorber at the same time during the synthesis of magnetite nanoparticles. Fe_3_O_4_ monodisperse nanocrystals with a saturation magnetization of 71.6 emu g^−1^ and a size of about 6 nm were produced, and the ionic liquid could be recovered for further usage.^73^ According to these findings, microwave irradiation can accelerate the development of magnetic phases in MIL-related materials while also encouraging the integration of ionic liquids or surface functionalization.

Microwave-assisted synthesis enables rapid processing and surface functionalization of magnetic IL-based materials. Cagli *et al.* (2024) reported IL-functionalized Fe_3_O_4_ nanoparticles with saturation magnetizations of 81 emu g^−1^ at 10 K and 73 emu g^−1^ at 300 K, maintaining colloidal stability for three months.^69^ Yadav *et al.* (2024) investigated hydrophobic IL-based magnetic adsorbents for water remediation.^70^ Fatahi *et al.* (2026) demonstrated microwave heating to 81 and 93 °C for [BIM][FeCl_4_] and [OIM][FeCl_4_], respectively, using 0.1 wt% aqueous dispersions at 400 W for 60 s.^72^ However, comparative data on synthesis yield, energy consumption, solvent use, production cost, and scalability remain limited. Therefore, claims of green synthesis, sustainability, and recoverability should be qualified until adequate techno-economic and safety evaluations are available.

#### 2.2.5. Electrochemical synthesis of metal-containing ionic liquids

This approach is not as important for completely replacing conventional MIL synthesis as it is for demonstrating electrochemical control of MIL behavior. The study showed that magnetic-field adjustment could improve the electrochemical reaction while preserving a wide electrochemical window. For MILs where simultaneous control of the metal oxidation state, redox activity, and ionic transport is necessary, electrochemical processing may be particularly beneficial.^2^

Gu *et al.* created a [FeCl_4_]^−^-based MIL with a functionalized imidazolium cation and showed that its electrochemical behavior could be controlled by an external magnetic field. Directional channels for Li^+^ and Cl^−^ migration were created *via* magnetic-field-induced microdomain creation and preferential alignment of MIL ions, which enhanced the electrolyte's electrochemical responsiveness. The higher capacitance and improved cycling stability of the magnetic-field-controlled electrolyte showed that the magnetic nature of a MIL can be directly linked to electrochemical ion movement.^54^

Jiang *et al.* used [BMIM]Cl FeCl_3_/[BMIM]Cl FeCl_2_ redox pair in a thermo-electrochemical cell to further illustrate the connection between magnetic MILs and electrochemical energy conversion. The system showed a thermopower of 0.26 mV K^−1^, an ionic conductivity of about 7 S m^−1^, and a charge-transfer resistance of 0.9 Ω cm^2^. An external magnetic field might control the diffusion of the paramagnetic Fe(iii)-containing component, allowing for the simultaneous conversion of mechanical and thermal energy into electrical power.^74^

Another important factor is cation coordination. Eight tetrachloroferrate ILs containing cations generated from pyridine, benzimidazole, trimethylamine, and triphenylphosphine were synthesized by Zafar *et al.* (2021). Their electrochemical measurements produced a heterogeneous electron-transfer rate constant of about 3.60 × 10^−3^ cm s^−1^ and a diffusion coefficient of about 7.55 × 10^−1^ cm^2^ s^−1^, suggesting that ion transport and interfacial electron-transfer kinetics can be tuned by altering cation structure.^19^ The potential of metal-containing ILs as redox-active electrolytes for flow-battery systems was further highlighted by the low-melting [C_4_py][FeCl_4_] described by Balischewski *et al.*, which showed reversible redox behavior in both aqueous and nonaqueous conditions.^75^ This method is more significant for showing electrochemical control of MIL behavior than it is for totally replacing traditional MIL synthesis. The work demonstrated that despite maintaining a broad electrochemical window, magnetic-field modification could enhance the electrochemical reaction.^10^ Therefore, electrochemical processing may be especially helpful for MILs where simultaneous control of the metal oxidation state, redox activity, and ionic transport is required.

#### 2.2.6. Biphasic extraction and ion-exchange methods

Because they enable the effective transfer of metal-containing species between aqueous and organic/ionic phases, biphasic synthesis and ion-exchange techniques continue to be crucial for the production of hydrophobic MILs. In 2024, a study was conducted that synthesized [P_66614_]_2_[CoCl_4_] and [P_66614_]_3_[DyCl_6_] and assessed them as extraction phases for five PAHs. While the Dy-based MIL needed 100 mg MIL and 10% NaCl, the ideal conditions were pH 2, 60 minutes of extraction time, 15 mg [P_66614_]_2_[CoCl_4_], and 7.5% NaCl. Over the course of five extraction cycles, the recovered Co-based MIL maintained >99% pyrene extraction.^76^

A geminal dicationic MIL created especially for tetracycline extraction was another invention in 2025. An aqueous biphasic extraction technique was utilized after the novel dicationic architecture was created to offer more robust interaction with the target analytes. Recyclability was shown experimentally, and the extraction mechanism was explained using density-functional-theory calculations.^77^ This signifies a change from merely swapping anions to logically designing the ionic structure in accordance with the target molecule.

A notable example of incorporating magnetic centers into both ionic components to achieve improved magnetic susceptibility while also lowering viscosity sufficiently for microextraction is the 2024 bimetallic MIL research. There was no need for a dispersive solvent or back-extraction phase; only 75 µL of MIL and five minutes of extraction time were needed. Enrichment factors were greater than 17, detection limits were 7.50 ng L^−1^, and inter-day RSD values were less than 11% (Fig. 5).^78^

**Fig. 5:**
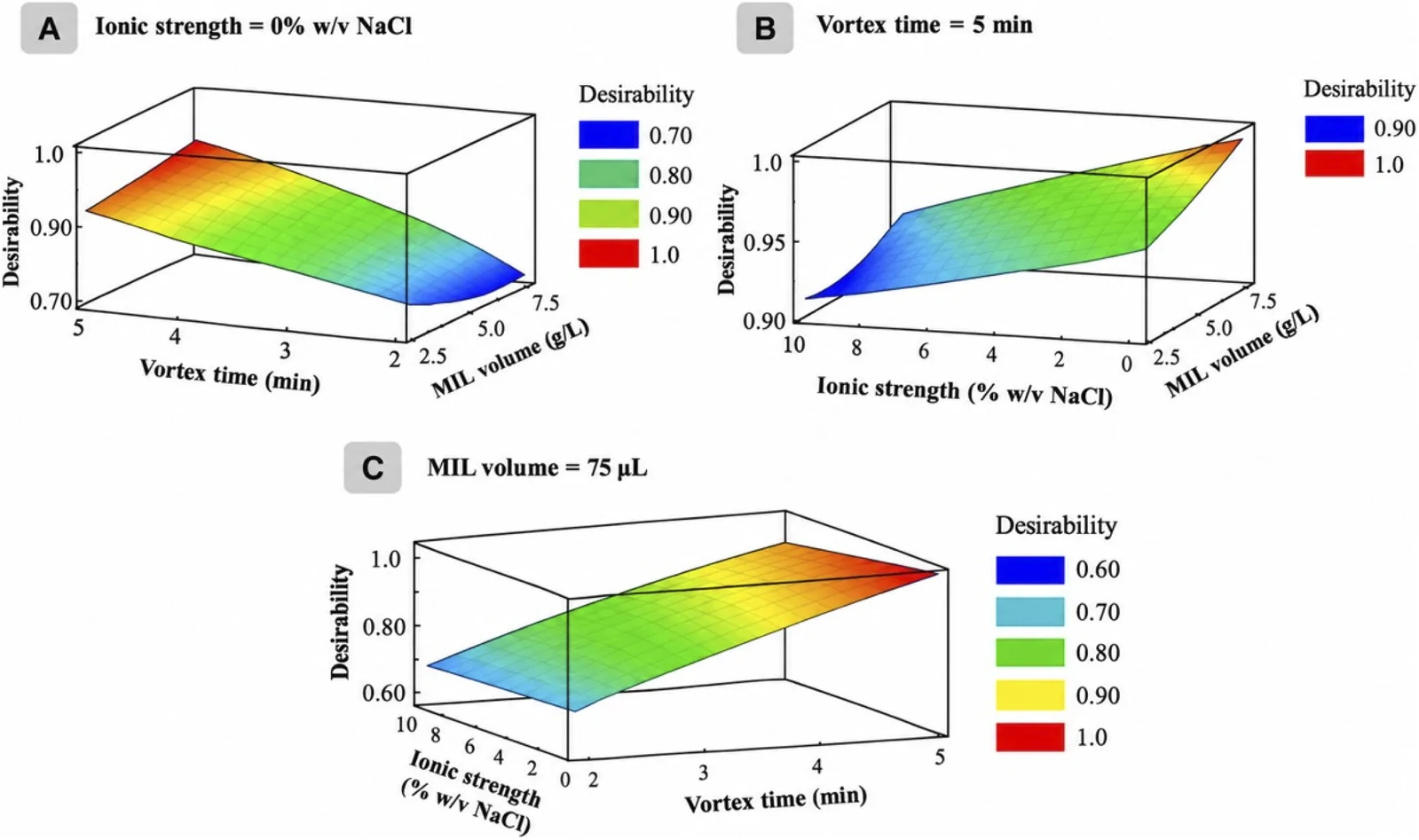
Response surfaces from the Box–Behnken design for the assessed OH-PAHs, with (A) ionic strength, (B) vortex time, and (C) MIL volume fixed at their optimum values.^78^

In the same way, the 2025 geminal dicationic MIL shows how changing the cation architecture can add more recognition sites without sacrificing the capacity to separate magnetically.^77^ Therefore, the ability to integrate multiple activities into a single material is the main benefit of such dual-functionalization. Nevertheless, adding more functional groups may also result in higher costs, viscosity, purification requirements, and synthesis procedures. Therefore, rather than being considered an intrinsic benefit, multifunctionality should be assessed in relation to the real improvement in application performance.^7^

According to recent studies, there is not a single synthetic path that is always better. Although mechanochemical processing provides scalable solid-state processing and lower solvent use, it is still not well-established for direct molecular MIL synthesis.^79^ Sol–gel techniques offer robust immobilization and recyclability at the expense of free MIL movement. While electrochemical methods provide options to manage redox-active metal centers, microwave processing can speed up synthesis and functionalization.^8^ While dual-functionalization and bimetallic designs are increasingly being employed to address the conventional trade-off between magnetic susceptibility and viscosity, biphasic and ion-exchange methods continue to be especially effective for hydrophobic extraction-oriented MILs.^16^ These new findings imply that, rather than focusing only on yield, future MIL synthesis should be assessed using a wider range of factors, such as reaction yield, solvent and energy consumption, viscosity, magnetic responsiveness, purity, scalability, recyclability, and application-specific performance.^26^

The main synthesis pathways for MILs are compiled in Table 2 and contrasted with respect to reaction conditions, synthesis time, quantitative performance, recyclability/stability, and practical benefits or drawbacks. It demonstrates how various techniques, such as solvent-free synthesis, sol–gel immobilization, microwave-assisted preparation, biphasic ion-exchange, MOF/coordination-polymer integration, and electrochemical methods, offer unique ways to regulate the magnetic, structural, catalytic, extraction, and electrochemical characteristics of MILs. Overall, the chart shows how traditional MIL preparation is giving way to quicker, solvent-minimized, recyclable, immobilized, and application-specific synthesis platforms; scalability and liquid-phase mobility retention are still crucial factors.

**Table 2:** MILs synthesis routes: conditions, performance and scalability

Synthesis route	Representative system	Conditions/time	Quantitative outcomes	Recyclability/stability	Advantage/limitation
Solvent-free/heat-stir	Co^2+^/Ni^2+^ [NTf_2_] MILs	<50 °C, ∼12 h; vacuum-dried 24 h; *in situ* in water <30 s	*µ*_eff_ 2.78–5.16 µB; viscosity as low as 198.3 cP; stable to 345 °C	High; no organic solvent or anion-exchange purification needed	Best for transition-metal chelate MILs; limited mechanochemical work^21^
Sol–gel/immobilization	Fe_3_O_4_@SiO_2_-imidazolium IL magnetic network	0.1 mol%, 85 °C, solvent-free; 10 reuse cycles	Magnetization ∼31.5 emu g^−1^; surface area 1177 m^2^ g^−1^; pore size ∼4.2 nm; ester yield 78–99%	High (10 cycles); pesticide recovery 82.4–109.8%; LOD 0.04–1.63 ng L^−1^	Immobilization sacrifices liquid-phase mobility for durability/recyclability^65^
Microwave-assisted	IL-functionalized Fe_3_O_4_ NPs	80 °C, single-step; stable in water/DMSO ∼3 months	Saturation magnetization: 81 emu g^−1^ (10 K), 73 emu g^−1^ (300 K); superparamagnetic	Moderate; IL controls particle size, surface, magnetism, colloidal stability	Fast synthesis; microwave heating *via* ionic conductivity & dipolar polarization^69^
Biphasic/ion-exchange	[P_66614_]_2_[CoCl_4_] and [P_66614_]_3_[DyCl_6_]	pH 2; 60 min extraction; 15 mg MIL; 7.5% NaCl	>99% pyrene extraction; 5 consecutive cycles; DFT-guided cation design	High for extraction-oriented MILs; bimetallic MILs: EF > 17, LOD 7.50 ng L^−1^, RSD < 11%	Most established for hydrophobic MILs; dual-functionalization boosts multifunctionality^76^
MOF/coordination polymer	IL@MIL-101(Cr); IL@MOF-526	Ship-in-bottle; grafting; *in situ* assembly	CO_2_ uptake 47.4 cm^3^ g^−1^ (273 K); BET 1162 m^2^ g^−1^; 92.5% carbonate yield; CO 14.0 mmol g^−1^ in 72 h (97% selectivity)	5–6 cycles reported; proton conductivity 1.28 × 10^−1^ S cm^−1^ at 60 °C/100% RH	Combines porosity, ionic character, catalytic sites, and magnetic separation^57,61^
Electrochemical	[FeCl_4_]^−^ MIL magnetic-field electrolyte	Magnetic-field-tuned directional ion migration	Electrochemical window ∼5.0 V; *χ* 1.82 × 10^−1^ emu g^−1^; thermopower 0.26 mV K^−1^	96.3% Coulombic efficiency (250 cycles); ionic conductivity ∼7 S m^−1^; *R*_ct_ 0.9 Ω cm^2^	Uniquely controls metal oxidation state + redox activity + ion transport simultaneously^54^

Although biphasic extraction and ion-exchange methods demonstrate promising performance, including >99% pyrene extraction over five cycles and enrichment factors above 17, these metrics do not establish overall sustainability. The geminal dicationic MIL also shows potential for tetracycline extraction through aqueous biphasic extraction and recycling. Thus, the Co-based MIL appears most promising when repeated extraction performance is the primary criterion, whereas the bimetallic MIL may be preferable for rapid, solvent-minimized analytical extraction. However, these materials cannot be ranked universally because they were evaluated for different target compounds and under different conditions. Moreover, extraction efficiency and reuse alone do not establish overall sustainability.

## 3. Physicochemical properties

The focus of recent research on MILs has switched from traditional Fe-based systems to low-viscosity bimetallic MILs, transition-metal chelate MILs, multifunctional magnetic electrolytes, and MILs dispersed with nanoparticles. These findings show that the identity and coordination environment of the magnetic center, as well as the structure of the organic and inorganic ionic components, have a significant impact on the physicochemical characteristics of MILs.^16^

### 3.1. Magnetic behavior and measurement techniques

Instead of merely raising the concentration of paramagnetic ions, recent MIL research has shown that the magnetic response may be controlled by the metal center and its coordination environment. A new family of transition-metal-based MILs including [Co(HIm)_6_][NTf_2_]_2_, [Co(OIm)_6_][NTf_2_]_2_, [Co(AmIm)_6_][NTf_2_]_2_, [Co(DMAc)_6_][NTf_2_]_2_ and [Co(DMP)_6_][NTf_2_]_2_, together with their Ni^2+^ analogs, was created in a 2024 study. The effective magnetic moments that were measured varied between 2.78 and 5.16 µB. In general, the Co-based systems had stronger magnetic responses than their Ni counterparts. For instance, [Co(OIm)_2_][NTf_2_]_2_ showed *µ*_eff_ = 4.47 µB, whereas [Ni(OIm)_6_][NTf_2_]_2_ showed 2.78 µB.^21^

In 2025, an alternative method utilizing [Bmim][FeCl_4_] comprising uncoated Fe_3_O_4_ nanoparticles was disclosed. In contrast to molecular MILs, this system's magnetic response may be directly related to rheological behavior. The suspension showed a significant decrease in relaxation time and an increase in viscosity toward a saturation value under an external magnetic field, suggesting a field-induced shift from primarily viscous to more elastic behavior. This illustrates the growing expansion of magnetic characterization of MIL-based systems from static susceptibility measures to field-dependent and magnetorheological measurements pertinent to engineering applications.^80^

### 3.2. Thermal and phase behavior

Heat capacity, viscosity, thermal breakdown, and temperature-dependent features were among the thermophysical characteristics of [C_4_mim][FeCl_4_] and [P_66614_][FeCl_4_] that Sanchez *et al.* examined. The comparison showed that although maintaining the same magnetic [FeCl_4_]^−^ anion, altering the cation significantly alters the thermal and phase-related properties.^81^ The thermal behavior and crystal formation of [C_2_mim][FeCl_4_] were described by Pardo *et al.* DSC revealed that MILs can have several solid phases prior to melting, with a solid–solid transition at 261 K, melting at 283 K, and disintegration above 623 K.^2^ Thermally induced polymorphism in [Dimim][FeCl_4_] was shown by de Pedro *et al.* (2016). Conversion to a high-temperature phase over 180 K was one of two consecutive solid–solid transitions that were found to be connected to the translational and reorientational motion of both ions. The significance of cooling rate and thermal history in MIL phase behavior is directly established by this investigation.^82^

When Murugappan *et al.* examined [3C_6_PC_14_][FeCl_4_], they discovered a single-stage breakdown process and thermal stability up to about 623 K (350 °C) under N_2_. The study shows that the useful thermal window of hydrophobic MILs is significantly influenced by cation architecture.^83^

Acid-functionalized [AcMIm][FeCl_4_] was investigated by Saha *et al.* (2017). TGA demonstrated stability up to about 230 °C, after which there were three stages of degradation with peaks close to 320, 482, and 597 °C. The various stages of decomposition show that the thermal degradation pathway can be significantly changed by functionalizing the organic cation.^84^

Abe *et al.* (2023) used simultaneous XRD and DSC to systematically analyze [C_*n*_mim]_2_ [MnCl_4−*m*_Br_*m*_] MILs. Anion configurational entropy and cation conformational entropy significantly influenced melting temperatures, while shorter alkyl chains and more symmetric anions promoted crystallization. This offers compelling evidence for composition-dependent phase behavior at the molecular level.^85^ Chang *et al.* (2022) investigated [C_2_mim][FeCl_4_] confinement in SBA-15. The bulk MIL showed melting at 287.5 K, solid–solid transition of 256.8 K, and cold crystallization at 235.2 K. Nanoscale confinement can significantly alter MIL phase transitions, as evidenced by the reduction of the melting temperature to 235.4 K in 5.39 nm pores and 249.4 K in 8.36 nm pores.^86^

Temperature-dependent phase equilibria of MIL aqueous two-phase systems based on PPG_400_ were examined by Yao *et al.* (2022). The systems demonstrated temperature-sensitive phase separation, with effective correlation utilizing empirical equations and experimentally recorded binodal curves. This shows that MIL phase behavior includes temperature-controlled liquid–liquid equilibria in addition to melting and crystallization.^87^

Chiral l-proline-based MIL aqueous two-phase systems at 298.15 K were also investigated by Yao *et al.* (2022). Phase equilibrium is regulated by both MIL structure and salt composition, as demonstrated by the experimental determination and correlation of tie-line/binodal data for three chiral MILs examined with various inorganic salts.^88^

Nie *et al.* (2024) examined three MILs based on cholinium in aqueous two-phase systems at various salt compositions and temperatures. The systems showed LCST-type phase behavior and the biphasic zone grew with temperature; the scientists mainly attributed phase separation to salt-induced salting-out and hydration effects. As a recent illustration of thermally changeable phase behavior in MIL systems, this is quite helpful.^89^

As a result, current research is moving away from reporting a single decomposition temperature and toward a comprehensive thermal operating window that incorporates melting/glass transition, crystallization, decomposition, viscosity, phase stability, and temperature-responsive functionality. Reliable comparison also necessitates taking into account the atmosphere, heating rate, sample history, water content, and the operational definition of decomposition temperature.^16^

### 3.3. Viscosity, density and conductivity

In MILs, the degree of ion association, temperature, alkyl chain length, and cation–anion structure all have a significant impact on viscosity, density, and ionic conductivity. Sanchez *et al.* demonstrated that cation shape significantly influences transport qualities by comparing [C_4_mim][FeCl_4_] and [P_66614_][FeCl_4_]. Compared to the imidazolium counterpart, the phosphonium MIL showed a density of about 0.99 g cm^−3^ and a significantly greater viscosity, indicating the significant impact of long alkyl chains on molecular packing and fluidity.^81^ Zhang *et al.* thoroughly examined mixed Fe–Cl/Br systems and 1-methyl-3-alkylimidazolium tetrachloroferrate MILs. The MILs had densities of 1.25–1.94 g cm^−3^, viscosities of 21–65 mPa s, and conductivities of 0.23–1.37 S m^−1^ at ambient temperature. The relationship between molecular structure and ion transport is demonstrated by the fact that increasing alkyl-chain length often increased viscosity while decreasing conductivity.^90^

Sanchez *et al.* used a group-contribution approach to investigate the temperature dependence of MIL viscosity. According to their analysis, viscosity significantly decreases with temperature, and the anion contribution is more important in explaining the temperature dependency than the cation contribution. In the absence of experimental data, this offers a helpful foundation for viscosity prediction.^91^ Daneshvar *et al.* (2020) examined [emim][FeCl_4_], [Bmim][FeCl_4_], and [Hmim][FeCl_4_] over 293.15–453.15 K using molecular-dynamics simulations. Alkyl-chain length, temperature, and ion transport were found to be related at the molecular level by the simultaneous evaluation of density, viscosity, self-diffusion, transport numbers, and electrical conductivity.^92^

The impact of magnetic fields on the rheological behavior of [P_66614_][FeCl_4_] was examined by Santos *et al.* Its viscosity dropped from roughly 749 mPa s in the absence of a magnetic field to 672 mPa s at 2 T, indicating that MIL viscosity may be externally adjusted by temperature, chemical structure, and magnetic field strength.^26^ Alkyl-chain effects in [C_*n*_C_1_Im][FeCl_4_] and [C_*n*_C_1_Im][FeCl_3_Br] were thoroughly investigated by Knoop *et al.* in 2022. The density of [FeCl_4_]^−^ MILs decreased from 1.381 to 1.230 g mL^−1^ when the chain was extended from C4 to C8, although the viscosity increased from 45.8 to 60.9 mPa s. Viscosities for [FeCl_3_Br]^−^ rose from 37.9 to 70.1 mPa s, indicating that longer hydrophobic chains lower density while increasing viscous resistance.^20^

Pierson *et al.* (2023) used functionalized diglycolic-acid ester ligands to create hydrophobic multi-metal MILs. The resulting MILs addressed the conventional trade-off between magnetic strength and fluidity by combining low viscosity with improved magnetic susceptibility. The work shows that viscosity can be decreased while maintaining many paramagnetic centers by ligand coordination.^16^

By Zhao *et al.* (2022), the density, viscosity, and ionic conductivity of BmimFeCl_4_ as an electrolyte were investigated as a function of temperature. While viscosity reduced as the temperature rose, density declined steadily between 20 and 80 °C. For charge transport in MIL-based electrolytes, the study emphasizes the significance of low viscosity and adequate ionic conductivity.^39^

In their investigation of an uncoated iron oxide nanoparticle dispersion in [BMIM][FeCl_4_], Sharma *et al.* (2025) found significant magnetorheological behavior. Shear thinning was seen in the MIL dispersion, and the application of a magnetic field decreased the relaxation time and raised viscosity toward a saturation value, indicating that magnetic interactions can dynamically modify viscoelasticity and MIL-based fluid movement.^80^

Transition-metal and lanthanide-containing MILs with viscosities ranging from roughly 198 mPa s to several thousand mPa s were reported in the 2022 multi-metal MIL study by Abbasi *et al.* These viscosities greatly rely on the metal center, ligand environment, and cation structure. Instead of handling viscosity and magnetic responsiveness separately, the study shows that coordination chemistry offers an efficient way to jointly tune these features.^24^ Together, these studies show that cation/anion selection, alkyl-chain engineering, metal–ligand coordination, temperature, and external magnetic fields can all rationally control the transport properties of MILs, offering a foundation for creating MILs with desired flow and charge-transport properties.

### 3.4. Tunable properties *via* composition control

By altering the cation, anion, metal center, ligand environment, and alkyl-chain length, MILs' physicochemical characteristics can be methodically adjusted.^14^ Three Mn-based fluorescent MILs with methodically altered alkyl-chain lengths were created by Huang *et al.* (2021). A reversible crystal-to-liquid-crystal transition linked to modifications in Mn-halide coordination was achieved when the cation structure was changed, changing the emission color from green/yellow to red. This shows that cation composition can control phase, optical, and magnetic behavior at the same time.^93^

Li *et al.* (2023) compared, [C_12_mim][FeCl_4_], [C_12_mim]_2_[CoCl_4_], and [C_12_mim]_2_[NiCl_4_], and all three MILs showed spontaneous adsorption on low-rank coal. Thus, the work shows that metal-center substitution offers a straightforward way to change MIL interfacial characteristics.^94^ Chand *et al.* (2019) systematically altered the *N*-alkylimidazole ligand (butyl *vs.* octyl) and the metal center (Ni^2+^, Co^2+^, and Mn^2+^). Effective magnetic moments varied with the metal center, and the resulting MILs had viscosities ranging from 152 to 2501 cP; Co(OIm)_4_(Cl)NTf_2_ was the least viscous at 152 cP. Therefore, independent control of magnetic and flow properties is possible by simultaneous alteration of metal and ligand.^95^

Morgado *et al.* (2021) used molecular modeling to examine [Bmim]^+^/[Emim]^+^ paired with [FeCl_4_]^−^, [FeBr_4_]^−^, [ZnCl_3_]^−^, and [SnCl_4_]^2−^. According to SAPT/DFT calculations, the metallic anion interacts preferentially close to the imidazolium ring's C2 region, and electrostatic and dispersion interactions are altered when the metal-halide composition is changed. This explains composition-dependent MIL characteristics at the molecular level.^44^ The alkyl-chain length of alkyl trimethyl ammonium-based MIL surfactants was modified in a 2021 magnetic-microemulsion investigation. The monophasic zone grew and the aggregation behavior changed as the chain length increased; the resulting W/O droplets were around 2–6 nm in size. The study shows that MIL self-assembly and interfacial behavior may be tuned *via* cation-chain engineering.^96^

With paramagnetic centers in both the cation and anion, Qiao *et al.* (2022) designed [Co(DMBG)_2_][Co(hfaca)_3_]. This dual-metal compound served as a hydrophobic extraction phase for five fluoroquinolones and exhibited strong magnetic responsiveness. The work shows that adding magnetic centers to both ionic components can produce multifunctional MILs with improved magnetic extraction and manipulation capabilities.^31^ In a 2021 *in situ* MIL-generation investigation, the following [Co(C_4_IM)_4_]^2+^, [Ni(C_4_IM)_4_]^2+^, and [Ni(BeIM)_4_]^2+^-based MILs. Metal/ligand substitution can be carried out directly during MIL formation rather than requiring the creation of a set solvent beforehand, as demonstrated by the ability to choose the MIL structure in accordance with extraction needs by altering the metal and ligand composition.^97^

Magnetic poly(ionic-liquid) systems made from alkyl-imidazolium FeCl_4_ units were studied by Li *et al.* (2018). Magnetic susceptibility was enhanced by increasing the FeCl_4_^−^ content, but molecular ordering and phase behavior were changed by increasing the length of the alkyl chain. The study shows that composition can be utilized to alter magnetic response in MIL-derived polymeric structures as well as to modify individual MIL molecules.^98^

The alkyl-chain length of [C_*n*_mim][FeCl_4_] was systematically changed in magnetic MIL/PVDF hybrid research while maintaining a constant MIL loading of 20 weight percent. While PVDF β-phase formation and porosity rose with increasing chain length, crystallinity, thermal stability, and magnetoionic response declined; the C_2_ analog had the highest magnetoionic coefficient of 1.06 ± 0.015 V cm^−1^ Oe^−1^. This proves unequivocally how macroscopic material properties can be influenced by cation composition.^8^ All of these findings point to metal identity, ligand chemistry, cation architecture, alkyl-chain length, anion composition, and metal-center distribution as important compositional factors for individually adjusting MILs' optical, transport, magnetic, thermal, and interfacial characteristics.

### 3.5. Environmental stability and biocompatibility

Environmental stability is receiving increasing attention because the low vapour pressure of MILs does not automatically make them environmentally benign. Alkyl-chain length, head-group chemistry, and cation structure can all have a significant impact on biological interactions, according to recent studies on IL toxicity.^14^ A 2024 high-throughput investigation of 45 ionic liquids revealed significant variations in cytotoxicity between structures and demonstrated that head-group structure and side-chain length influenced the hazardous mode of action. Despite not being limited to MILs, this study offers a valuable framework for evaluating functionalized MILs because their biological interactions are governed by similar structural characteristics.^99^

The development of MILs for gene delivery and nucleic acid nanotechnology in the future will benefit greatly from this mechanistic insight. In human skin fibroblasts and Caco-2 cells, Frade *et al.* (2013) assessed MILs containing imidazolium/cholinium cations with [FeCl_4_]^−^, [GdCl_4_]^3−^, [CoCl_4_]^2−^, and [MnCl_4_]^2−^. According to the study, the magnetic anion had a significant impact on toxicity, with MILs containing Co and Mn having more cytotoxic effects.^100^

Sintra *et al.* (2017) used *Vibrio fischeri* to study cholinium-based MILs. The toxicity of all studied MILs ranged from mild to high, depending on the metal in the anion, the length of the alkyl chain, and the amount of hydroxyethyl groups. [MnCl_4_]^2−^ shown relatively low toxicity among the studied magnetic anions. Significant structure-dependent variations in cytotoxicity were found in a 2024 high-throughput investigation of 45 ILs.^101^

A recent comprehensive cytotoxicity dataset further demonstrated that longer alkyl chains (≥C6) were a major determinant of cytotoxicity for 3-alkyl-1-methylimidazolium ILs, although anion identity could also significantly modify toxicity.^102^ Both side-chain length and head-group structure influenced toxic mode of action, supporting the significance of molecular design when developing functionalized MILs.^99^

A molecular explanation for the structure-dependent toxicity shown in ILs has been provided by mechanistic investigations that have connected longer alkyl chains with stronger membrane contacts and disruption.^103^ Cationic alkyl-chain length and biocompatibility are strongly correlated, according to more recent biomedical research, with long-chain ILs exhibiting significantly higher cellular toxicity than short-chain counterparts.^104^

Aqueous persistence, biodegradability, cation/anion structure, and toxicity to aquatic creatures must all be taken into account, as larger environmental studies demonstrate that minimal vapor pressure does not guaranty environmental benignity.^105^ Instead of using non-volatility as a measure of environmental safety, a 2026 review highlights that IL safety assessment should incorporate cation/anion structure, experimental toxicity, biodegradation, and computational techniques.^106^

## 4. Applications of magnetic ionic liquids

### 4.1. Catalysis and reaction media

MILs, which combine the low volatility and structural tunability of ionic liquids with quick magnetic separation, are being developed more as magnetically recoverable reaction medium and catalysts (Table 1). According to recent research, the magnetic component can be immobilized on Fe_3_O_4_/SiO_2_, graphene oxide, mesoporous silica, or polymeric supports, or it can be integrated directly into the ionic phase. Catalyst recovery and reuse are made possible by the magnetic phase, while the cation/anion can regulate acidity, basicity, polarity, or metal coordination.^107^

The scope of this method is demonstrated by several recent investigations. In 2025, solvent-free esterification was catalyzed at just 0.1 mol% and 85 °C using a sulfonic acid-functionalized magnetic ionic network with silica-coated iron oxide and imidazolium poly (ILs) linkers. This resulted in 78–99% ester yields and maintained activity for ten cycles. The hydrophobic network enhanced water removal and reactant diffusion, showing how MILs architecture can enhance catalytic activity and separation at the same time.^66^

1,8-Dioxodecahydroacridines were synthesized in a single pot using a 2024 Fe_3_O_4_@SiO_2_-supported dicationic ionic-liquid catalyst (Fe_3_O_4_@SiO_2_·DIL). The catalyst demonstrated the advantage of dicationic functionality for multicomponent reactions by producing excellent yields with fast reaction times and being magnetically recovered without appreciable activity loss.^108^ Under ultrasonic irradiation, another 2024 Fe_3_O_4_@SiO_2_@CPTMS-DTPA magnetic ionic nanocatalyst facilitated the three-component synthesis of α-amino-phosphonates, yielding 64–97% yields in 10–25 minutes and remaining reusable for five cycles. In this case, the response was significantly shortened by both magnetic recovery and ultrasound-assisted mass transfer.^109^

The 2022 *n*-Fe_3_O_4_@SiO_2_-TA-SO_3_H IL catalyst showed the efficiency of immobilized acidic MIL functionality for multicomponent synthesis. One 2-hydroxy substrate reached 96% yield in about 10 minutes, and typical products in Hantzsch reactions were produced in 82–96% yield. Instead of using traditional filtration, the catalyst could be eliminated magnetically. Rather than only magnetism, this performance results from the high density of –SO_3_H groups in conjunction with the porous magnetic support.^110^

Additionally, MILs have been successful in metal-catalyzed coupling processes. For the model Heck coupling, a 2022 Fe_3_O_4_@SiO_2_@IL-PMO/Pd nanocatalyst produced 98% yield in 30 minutes with just 0.48 mol% Pd catalyst. While less reactive aryl chlorides still yielded 85–89%, aryl bromides typically yielded 95–97%. Reuse was possible for four cycles without significant performance degradation thanks to magnetic recovery. Pd is trapped in the mesoporous ionic-liquid shell, and separation is made possible by the Fe_3_O_4_ core.^111^

A 2022 WO_4_@Fe_3_O_4_/GO-IL catalyst coupled tungstate active sites with a magnetic ionic-liquid/graphene-oxide framework for the conversion of sulfide to sulfoxide in selective oxidation. While the comparable WO_4_@Fe_3_O_4_/GO material without the ionic-liquid component performed significantly worse, the catalyst was magnetically recovered and reused for five cycles with no discernible loss of activity or selectivity.^112^

In an H_2_O_2_-assisted system, the Fe-based MIL [HDMF]Cl_2_/FeCl_3_ served as both an extractant and a catalyst for oxidative desulfurization. Dibenzothiophene removal attained 98.38% at 60 °C for 100 minutes with 1.0 g MIL and an O/S ratio of 5. Because the same MIL phase concentrates the sulfur-containing substrate and encourages its oxidation, this dual extraction–catalysis activity is especially beneficial.^113^

Another significant use is the manufacturing of biodiesel. The [NiFe_2_O_4_@BMSI]HSO_4_ magnetic IL catalyst produced a biodiesel yield of 86.4% as opposed to 74.6% for its bromide counterpart, indicating that the counter-anion has a significant impact on catalytic acidity. After six cycles, both catalysts continued to exhibit significant activity.^114^ After six uses, a similar Fe_3_O_4_@SiO_2_@PIL catalyst with dual acidic IL activity achieved 92.1% oleic-acid conversion/methyl-oleate yield with minimal loss; its 14.3 emu g^−1^ magnetization allowed for quick magnetic separation.^115^

More recently, CO_2_-mediated aziridine conversion was carried out using a Fe_3_O_4_@SiO_2_@PIL-CeCl_4_ catalyst, which produced 87–95% isolated yields with 98.0–99.2% selectivity at 90 °C and 1 atm CO_2_. The system shows how a Lewis-acid metal center combined with IL activity can both improve catalyst recovery and encourage substrate activation.^116^

In 2023, alkaline imidazolium ionic-liquid polymer brushes on magnetic polymer microspheres, [Im][OH]/MP-B, were used to illustrate an alternative design concept. The quasi-homogeneous architecture is appealing for tandem 4*H*-pyran/biodiesel conversions because it combines the recoverability of a heterogeneous catalyst with the accessibility of a soluble catalyst.^117^

Recent application-focused advancements in MIL-based functional systems, including catalysis, extraction and separation, environmental remediation, biomedical applications, smart materials, energy storage, gas capture, and tribology, are shown in Table 3. It illustrates how the inherent combination of magnetic responsiveness, ionic character, tunable chemical functionality, and simple magnetic recovery allows MILs to function as multifunctional materials by comparing various MIL platforms based on their target process, quantitative performance, recyclability, and principal functional advantages. High catalytic yields, effective pollutant and biomolecule extraction, quick magnetic separation, therapeutic and detecting functions, improved electrochemical performance, and magnetically controlled lubrication are all demonstrated by the provided results. Overall, the table shows how MILs have evolved from mostly magnetic liquid systems to adaptable, recyclable, and application-specific materials for cutting-edge energy, chemical, environmental, and biological applications.

**Table 3:** Recent MILs-based catalysts, their representative catalytic reactions, performance metrics, and principal advantages

Application domain	MIL/system	Target reaction/process	Quantitative metrics	Recyclability	Advantage/insight
Catalysis	Fe_3_O_4_@SiO_2_ ionic network	Esterification	78–99% yield; 0.1 mol%; 85 °C; solvent-free	10 cycles	Sulfonic acid + mesoporosity + magnetic separation = sustainable acid catalysis^66^
Catalysis	Fe_3_O_4_@SiO_2_@IL-PMO/Pd	Heck coupling	98% yield, 30 min, 0.48 mol% Pd; ArBr 95–97%; ArCl 85–89%	4 cycles	Pd stabilized in mesoporous IL shell; Fe_3_O_4_ core enables separation^111^
Catalysis	[HDMF]Cl_2_/FeCl_3_ MIL	Oxidative desulfurization	98.38% DBT removal; 60 °C, 100 min; O/S ratio = 5; 1.0 g MIL	5 cycles	Same MIL concentrates substrate and catalyzes oxidation: dual function^113^
Catalysis	Fe_3_O_4_@SiO_2_@PIL-CeCl_4_	CO_2_/aziridine conversion	87–95% yield; 98.0–99.2% selectivity; 90 °C, 1 atm CO_2_	4 cycles	Lewis acid Ce + IL activity = enhanced substrate activation + easy recovery^116^
Extraction/separation	[P_66614_]_2_[CoCl_4_] + [P_66614_]_3_[DyCl_6_]	PAH extraction from water	>99% pyrene; 15 mg MIL; pH 2; 7.5% NaCl; 60 min	5 cycles	Bimetallic MILs avoid dispersive solvent; EF > 17; LOD 7.50 ng L^−1^ (ref. 76)
Extraction/separation	[N_1,8,8,8_][FeCl_4_]	Cd/Zn chlorocomplex extraction	99.7% Cd; 81.0% Zn at 3.3 mg mL^−1^ MIL, 30 min; no centrifugation	5 cycles	Magnetic phase recovery eliminates centrifugation in saline matrices^36^
Extraction/separation	OSMIL (oligonucleotide-MIL)	Sequence-specific DNA extraction	∼897 nm particles; magnetic recovery in ∼3 s; RSD = 1.19% (*n* = 3)	5 cycles	Combines molecular recognition and magnetic manipulation in one material^33^
Environmental	[P_66614_][FeCl_4_] effervescence-MIL	Hg speciation in water/wine	75 µL MIL; no centrifugation; rapid magnetic separation	4 cycles	AFS detection of inorganic/organic Hg; effervescence-assisted dispersal^35^
Environmental	IL-modified FeO NPs	Crystal violet dye + Cr(vi) removal	Improved over unmodified FeO NPs; hydrophobic IL coating	Recyclable	Hydrophobic functionalization enhances pollutant affinity^70^
Environmental	[C_16_mim][FeCl_4_] microemulsion	Oily sludge treatment	Residual oil reduced to 1.36% after 5 cycles; MIL maintained effectiveness	5 cycles	Combines MIL surfactancy with magnetic recovery^128^
Biomedical	MIL-carbon nanohorn nanocomplex	Cancer theranostics (photothermal)	Photothermal efficiency 63%; stable in water ∼7 days; NIR fluorescence + magnetic guiding	Moderate	[Bmim][FeCl_4_] + ICG + CNH—combines 4 modalities in one platform^37^
Biomedical	Magneto-hydrogel (valine-MIL surfactant)	Drug delivery	Encapsulation: 69 ± 0.6% ornidazole; 78 ± 0.3% 5-fluorouracil; magneto-responsive gelatin	Good	MIL replaces conventional magnetic nanoparticles in hydrogel actuation^126^
Smart materials/devices	[Bmim][FeCl_4_]/P(VDF-TrFE) composite	Magnetoelectric actuator/sensor	Bending: 2.1 mm at ∼3 kOe; ME coeff ∼15 V cm^−1^ Oe^−1^; sensitivity 76 mV Oe^−1^; 40 wt% MIL	Flexible, stable	Single material combines sensing, actuation, ionic conductivity; no separate magnetic filler^119^
Energy/electrochemistry	IL electrolyte (Li/LFP cells)	Li-ion battery electrolyte	96.3% coulombic efficiency (250 cycles); 93.2% capacity retention (150 cycles)	250 cycles	Addresses viscosity–conductivity trade-off in next-gen IL electrolytes^121^
Energy/electrochemistry	[N_1114_][NTf_2_] supercapacitor	High-voltage supercapacitor	∼2000 F g^−1^ at 2.6 A g^−1^; stable up to 3.6 V	Stable	IL-type electrolyte extends operating voltage window for energy storage^122^
Gas capture/separation	Silica-immobilized dicationic IL (FeCl_4_^−^)	CO_2_ capture	57.31 ± 0.02 mg CO_2_ g^−1^; CO_2_/N_2_ selectivity ×3.8 *vs.* pure silica	Recyclable	Dual-cation structure with magnetic anion simultaneously enhances CO_2_ affinity^129^
Tribology	[Bmim][FeCl_4_] in CF10W-40 lubricant (2 wt%)	Lubrication additive	Friction ↓20%; wear depth ↓45%; 0.1 T field: friction ↓10% more, wear ↓20% more; critical load ↑20% at 1 T	Stable additive	First quantitative demonstration of magnetically controlled lubrication with MIL^131^

Collectively, these results demonstrate that multifunctionality, not just magnetism, is the true benefit of MIL catalysis. While MILs add magnetic separation, conventional homogeneous ILs offer superior reaction conditions but are challenging to recover; conventional solid catalysts are easily separated but provide less control over the ionic microenvironment. Before MIL catalysts can be regarded as truly sustainable at industrial scale, future research should focus on metal/IL leaching, catalyst lifetime over tens to hundreds of cycles, continuous-flow operation, solvent and energy consumption, and techno-economic assessment.^107^

### 4.2. Separation and extraction processes

MILs are particularly attractive for smart-material applications because their magnetic response is intrinsic to the ionic liquid and does not require a separate magnetic-particle phase. This gives MILs a major advantage over conventional magnetorheological fluids, in which magnetic particles are dispersed throughout a carrier liquid and may undergo sedimentation, phase separation, or aggregation.^26^ According to recent research, the transport and rheological behavior of MILs can be directly altered by an external magnetic field. For instance, the viscosity of [C_4_C_1_im][FeCl_4_] decreased by 10.3% at 2 T, whereas the viscosity of [C_8_C_1_im][FeCl_4_] decreased by 20.1% at 2 T along with increased molecular diffusion.^118^ For microvalves, pumps, dampers, and adaptive fluidic systems, such reversible field-dependent viscosity and diffusion offer a way to regulate fluid resistance, flow rate, and mass transport without mechanical contact.

Adding magnetic nanoparticles to a MIL matrix can increase the responsiveness even more. Fe_2_O_3_/Fe_3_O_4_ nanoparticles in [Bmim][FeCl_4_] showed significant magnetorheological behavior in a 2025 study, with changes in viscosity and viscoelasticity brought on by magnetic fields. Crucially, although similar particles exhibited sedimentation in the non-magnetic [Bmim][BF_4_] medium, the Fe_3_O_4_ particles stayed uniformly distributed in the MILs.^80^ Therefore, when a greater magnetic response is needed than what a molecular MIL alone can deliver, this hybrid approach can be helpful.

When integrated into flexible polymers, MILs can transform magnetic stimulation into mechanical deformation for soft actuators and artificial muscle-type devices. MILs can combine magnetic inputs with electrical and mechanical outputs in a flexible device, as proven by a composite based on [Bmim][FeCl_4_]/P(VDF-TrFE) that displayed both magnetic sensing and bending-actuation capabilities.^119^ When compared to traditional magnetic fillers, this multifunctionality is especially appealing because the MIL concurrently offers processability, magnetic responsiveness, and ionic conductivity, eliminating the requirement for several functional additives.

Because tiny variations in magnetic field can result in detectable changes in viscosity, diffusion, electrical response, or mechanical deformation, MILs are also intriguing for magnetic-field sensors and microfluidic devices. Valves, pumps, actuators, dampers, and robotic systems are already supported by conventional magnetorheological materials; however, MILs provide a liquid, electrically active substitute that may be incorporated into smaller channels.^120^ Compact lab-on-a-chip systems benefit from their inherent magnetic nature, which also permits magnetic manipulation and phase separation without centrifugation.^23^

As a result, the main benefit of MILs is their multifunctionality: a single liquid can offer chemical activity, ionic conductivity, magnetic responsiveness, and adjustable viscosity. However, MIL-nanoparticle hybrids and MIL-polymer composites are emerging as more useful designs because pure MILs often have weaker magnetic responses than particle-based magnetorheological fluids. Therefore, rather than just enhancing magnetic susceptibility, future devices should concentrate on optimizing magnetic-field sensitivity, response/recovery time, viscosity, conductivity, cycling stability, and downsizing.^80^

### 4.3. Electrochemical and energy applications

MILs are appealing as functional electrolytes rather than just passive solvents because of their ionic mobility, magnetic responsiveness, and electrochemical stability. In order to address the viscosity–conductivity trade-off, recent research has increasingly concentrated on regulating ion transport and electrode–electrolyte interfaces. One 2024 electrolyte, for instance, retained 96.3% coulombic efficiency after 250 cycles and 93.2% capacity retention after 150 cycles in Li/LFP cells, demonstrating how contemporary IL electrolyte systems have enhanced conductivity while maintaining stable Li plating/stripping.^121^

Ionic-liquid electrolytes can raise energy density in supercapacitors by extending the operating voltage. A 2025 investigation with [N_1114_][NTf_2_] showed a specific capacitance of about 2000 F g^−1^ at 2.6 A g^−1^ and stable operation up to 3.6 V. These findings demonstrate the promise of MIL/IL-type electrolytes for high-voltage energy-storage devices, despite the fact that ion mobility and viscosity continue to be significant constraints.^122^

Additionally, MILs offer potential for electrochemical synthesis and electrodeposition, where magnetic fields can affect mass transport and ion organization close to electrodes. Additionally, their adjustable metal-containing anions offer a way to directly add electroactive species to the electrolyte, which may allow for the controlled deposition of catalytic or magnetic coatings. Recent ILs research in fuel cells shows that modifying cation–anion combinations can simultaneously enhance thermal stability and proton/ion transport; hydroxylammonium ionic liquids containing triflate, for instance, demonstrated conductivities of 58–64 mS cm^−1^ at 150 °C.^123^

Because its composition may be adjusted to regulate charge transfer, redox chemistry, and electrode interfaces, MILs are being investigated as non-volatile electrolytic media and interfacial materials for solar and photoelectrochemical systems. According to recent research, the next step is to build magnetic electrolytes with optimal conductivity, viscosity, electrochemical window, and interfacial compatibility for particular energy devices rather than just switching out traditional electrolytes with MILs. *In situ* electrochemical measurements in conjunction with molecular simulations are becoming crucial instruments for accomplishing this structure–property control.^124^

### 4.4. Biomedical applications

Transition-metal MIL surfactants comprising [FeCl_3_Br]^−^ have drawn interest in magnetically controlled drug delivery because the Fe-containing anion provides magnetic responsiveness while the long-chain cation offers self-assembly and drug/DNA interaction. More sophisticated devices obtain dual stimuli response by combining MILs with polymers or biomolecules. For instance, C60@CTAF/DNA, which incorporates the MIL [CTAB][FeCl_3_Br], was created as a magnetic nucleic acid/drug delivery platform; the addition of a hyaluronic acid coating brought glutathione-responsive behavior and made it possible to target liver cancer cell models.^125^ MILs can provide magnetic actuation without traditional magnetic nanoparticles, as demonstrated by an earlier but quantitatively useful magneto-responsive hydrogel based on a valine-derived magnetic ionic-liquid surfactant/gelatine network that achieved encapsulation efficiencies of 69 ± 0.6% for ornidazole and 78 ± 0.3% for 5-fluorouracil.^126^

An oligonucleotide strand-attached magnetic ionic liquid (OSMIL) for sequence-specific DNA extraction was described by Chen *et al.* (2023), as shown in Fig. 6. The target DNA was selectively recognized by the complimentary oligonucleotide, and extraction and magnetic manipulation were made possible by the hydrophobic MIL. During separation, the OSMIL transitioned from a liquid extraction phase to a solid magnetic phase, enabling target DNA recovery under an external magnetic field in about three seconds. It demonstrated good repeatability (RSD = 1.19%, *n* = 3), was reusable, and preferentially extracted DNA from mixes comprising other DNA sequences and proteins. Additionally, the technique was shown to extract target eDNA from various water conditions, suggesting promise for environmental DNA study and molecular identification.^33^

**Fig. 6:**
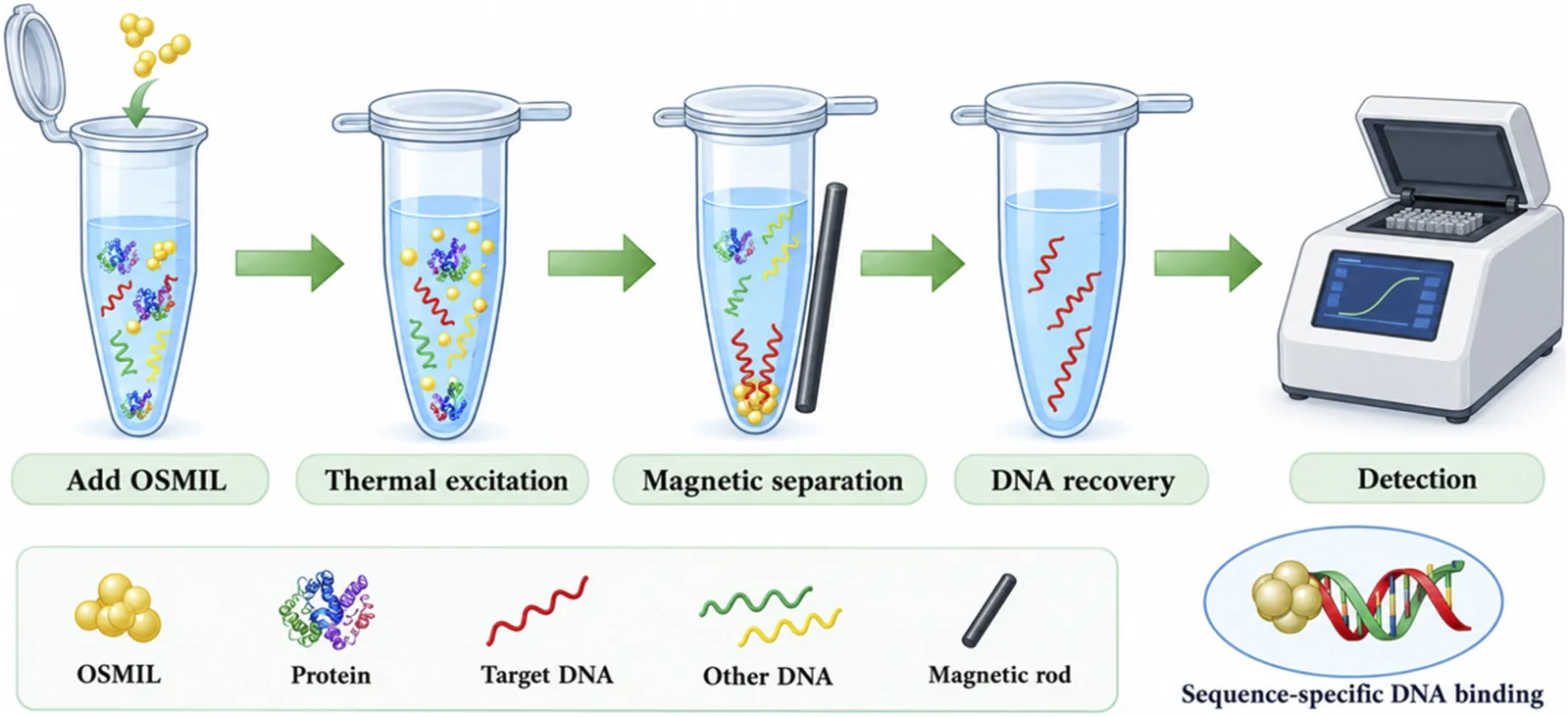
Schematic representation of sequence-specific DNA extraction using OSMILs, reproduced from ref. 33 with permission from Elsevier, *Anal. Chim. Acta*, 2023, **1239**(1), 340612, copyright 2022.

Additionally, MILs are being researched for therapeutic and MRI applications. The potential to combine imaging and therapeutic delivery in a single material has been demonstrated by paramagnetic surface-active MILs containing [FeCl_3_Br]^−^, especially long-chain imidazolium, pyridinium, and isoquinolinium derivatives, which have demonstrated both *T*_1_/*T*_2_ MRI contrast behavior and biomolecular interaction capability. Their main appeal over traditional Gd-based contrast agents is the potential to replace a distinct contrast agent/carrier combination with a structurally customizable, magnetically responsive platform.^127^ However, because there are still little comprehensive *in vivo* pharmacokinetic and long-term toxicity data, claims of better biocompatibility are still premature.

### 4.5. Environmental applications and remediation

MILs are appealing for the removal and recovery of pollutants because they combine the tunable solvation and extraction characteristics of ionic liquids with magnetic-field-assisted separation. Recyclable extraction of hazardous metals and organic contaminants has received special attention in recent research.

Five polycyclic aromatic hydrocarbons (PAHs) were extracted from water in 2024 using [P_66614_]_2_[CoCl_4_] and [P_66614_]_3_[DyCl_6_]. With >99% pyrene extraction, the Co-based MIL could be recovered and used again for five cycles in a row.^76^ For metal pollutants, magnetic microextraction of Cd and Zn chloro-complexes from saline water was made possible by [N_1,8,8,8_][FeCl_4_]. At 3.3 mg mL^−1^ MIL and 30 min extraction time, 99.7% Cd and 81.0% Zn separation were achieved, with magnetic recovery avoiding centrifugation.^36^

MILs can serve as magnetic sorbents or recyclable extractive phases, enabling quick separation following contamination capture. When compared to unmodified FeO nanoparticles, a 2024 hydrophobic IL-modified magnetic FeO system demonstrated improved removal of crystal violet and Cr(vi).^70^ Similarly, after 5 cycles, the MIL maintained its high oil-removal effectiveness, while a 2024–2025 MIL-based microemulsion employing [C_16_mim][FeCl_4_] for oily-sludge treatment reduced residual oil to 1.36%.^128^

According to recent computer research, MIL anions have a significant impact on gas affinity. MD simulations of MILs with Fe, Mn, and Gd magnetic anions based on [C_4_C_1_im]^+^- and [P_66614_]^+^ revealed advantageous solvation of CO_2_ and SO_2_, especially for multivalent anions.^45^ In an experiment, at 1 bar and 25 °C, a silica-immobilized magnetic dicationic IL containing FeCl_4_^−^ produced 57.31 ± 0.02 mg CO_2_ g^−1^, whereas CO_2_/N_2_ selectivity rose almost 3.8 times compared to pure silica.^129^

The combination of MILs' little volatility, adjustable chemical activity, and magnetic recoverability, which can minimize solvent loss and streamline phase separation, is their main environmental benefit. Low volatility alone, however, does not guaranty “green” performance; ionic-liquid toxicity, biodegradation, metal leaching, synthesis energy, and end-of-life recovery must all be taken into account. Therefore, prior to widespread environmental deployment, recent evaluations place a strong emphasis on recyclability, life-cycle assessment, toxicity testing, and the creation of less harmful cation/anion combinations.^130^

The environmental stability and biocompatibility of magnetic ionic liquids (MILs) depend on the combined effects of their organic cations, ligand structures, and paramagnetic metal-containing anions (Table 4). Frade *et al.* evaluated imidazolium- and cholinium-based MILs in human skin fibroblasts and colorectal adenocarcinoma cells, reporting greater cytotoxic potential for Co- and Mn-containing anions than for the Fe- and Gd-based systems tested.^100^ Similarly, Sintra *et al.* found that all investigated cholinium-based MILs exhibited moderate to high toxicity toward *Vibrio fischeri*, with toxicity influenced by cation side-chain length, hydroxyethyl-group number, and metal identity; the Mn-containing anion was the least toxic among those examined.^101^ These findings demonstrate that the use of a potentially biocompatible cation does not necessarily ensure that the complete MIL is environmentally safe. Although ligand structure can influence MIL properties, its independent contribution to toxicity remains insufficiently characterized. Therefore, systematic toxicity testing, biodegradability assessment, metal-leaching analysis, and evaluation across multiple organisms are essential for developing safer MIL formulations.^100,101,105,130^

**Table 4:** Representative cytotoxicity and ecotoxicity findings for MILs

MIL family or component	Reported findings	Environmental implication
Imidazolium-based MILs	Cytotoxicity varied with the magnetic anion; Co- and Mn-containing systems showed greater cytotoxic potential than Fe- and Gd-containing systems tested	Cation selection alone is insufficient to predict safety^100^
Cholinium-based MILs	All tested formulations were moderately toxic or toxic to *Vibrio fischeri*	Cholinium-based formulations should not automatically be considered environmentally benign^101^
Paramagnetic metal-containing anions	Co- and Mn-containing anions showed greater cytotoxic potential in the tested human cell lines	Toxicity depends on the biological test system and specific MIL composition^100,101^
Organic cation and structural groups	Alkyl side-chain length and the number of hydroxyethyl groups influenced ecotoxicity	Structural modification may help guide the design of less-toxic MILs^101^
Organic ligands and complete MIL formulations	Their independent contributions to toxicity remain insufficiently established in the cited studies	Further component-specific testing and environmental-fate assessment are needed^101,105^

### 4.6. Smart materials and devices

MILs are evolving into field-responsive smart materials because magnetic fields can change their molecular structure, viscosity, ion transport, and interfacial behavior without changing their chemical composition. This response is especially helpful for small devices that allow for remote control of fluid properties. For instance, at 2 T, [C_4_C_1_im][FeCl_4_] and [C_8_C_1_im][FeCl_4_] displayed viscosity drops of 10.3% and 20.1%, respectively, indicating that cation structure and alkyl-chain length can be used to adjust magnetic-field sensitivity.^118^

MILs can be integrated into flexible polymers to combine magnetic stimulation with electrical and mechanical responses for soft actuators and artificial muscle-like devices. A [Bmim][FeCl_4_]/P(VDF-TrFE) composite with 40 weight percent MIL showed a bending displacement of 2.1 mm in a magnetic field of about 3 kOe, a maximum magnetoelectric voltage coefficient of around 15 V cm^−1^ Oe^−1^, and a magnetic-field sensitivity of 76 mV Oe^−1^. As a result, the material offers advantages over traditional magnetic fillers that mainly provide mechanical magnetic response by combining sensing and actuation into a single flexible platform.^119^

Combining MILs with magnetic nanoparticles can result in a stronger magnetorheological reaction. Uncoated nanoparticles in a 2025 [Bmim][FeCl_4_]-Fe_2_O_3_/Fe_3_O_4_ system settled in the non-magnetic [Bmim][BF_4_] medium while being uniformly distributed in the MIL. Under an applied magnetic field, the MIL–nanoparticle fluid showed shear-thinning behavior that gradually decreased; its viscosity increased toward a field-dependent saturation value, while the relaxation time decreased, suggesting a change from a more viscous to a more elastic response. Because of this, the technology is appealing for magnetically controlled fluidic devices, soft robotics, and adaptive dampers. The [Bmim][FeCl_4_]/P(VDF-TrFE) system's exhibited 76 mV Oe^−1^ sensitivity and 2.1 mm actuation displacement show how sensing and actuation can be combined into a single flexible material.^80^

Magnetic responsiveness offers contactless control and quick phase manipulation for microfluidics and lab-on-a-chip systems. With just 75 µL of MIL, a 2024 MIL-based effervescence-assisted microextraction method utilizing [P_66614_][FeCl_4_] allowed for quick magnetic separation without centrifugation for the extraction of mercury species. Although the comparatively high viscosity of certain MILs continues to be a barrier for high-speed flow, such behavior is beneficial for automated microfluidic extraction, magnetic valves, and compact sample-processing devices.^35^

### 4.7. Miscellaneous applications

MILs are finding new uses in tribology, electromagnetic-wave attenuation, sophisticated composites, and gas separation in addition to environmental cleanup, energy storage, catalysis, medicinal systems, and smart gadgets. Magnetically controlled lubrication is a highly promising field. When [Bmim][FeCl_4_] was added to CF10W-40 lubricant at a weight percentage of 2 weight percent in 2024, the additive decreased wear depth by 45% and friction by around 20%. Applying a 0.1 T magnetic field further reduced wear by 20% and friction by roughly 10%. The boundary film's critical load increased by around 20% at 1 T, indicating that MILs can provide the interfacial film with both lubrication and magnetic control.^131^

Additionally, selective gas capture and separation using MILs is being investigated. Imidazolium- and phosphonium-based MILs comprising [FeCl_4_]^−^, [FeBr_4_]^−^, [MnCl_4_]^2−^, and [GdCl_6_]^3−^ were investigated in 2025 MD research for interactions with CO_2_, SO_2_, CH_4_, and NH_3_. The paper provides a computational path toward task-specific MILs for CO_2_ capture and industrial gas separation by showing how adjusting the magnetic anion and cation can modify gas solvation and transport.^45,132^

These less well-known uses show that MILs are developing into magnetically controlled lubricants, electromagnetic-absorbing composites, and selective gas-separation media in addition to traditional extraction solvents.^133–136^ While gas-separation applications are still relatively exploratory and require additional experimental validation, long-term stability studies, and techno-economic assessment prior to practical deployment, tribological and electromagnetic applications already have quantitative experimental validation. Fig. 7 summarizes the key milestones in MIL development, highlighting the evolution from early ionic liquids to emerging functional applications.

**Fig. 7:**
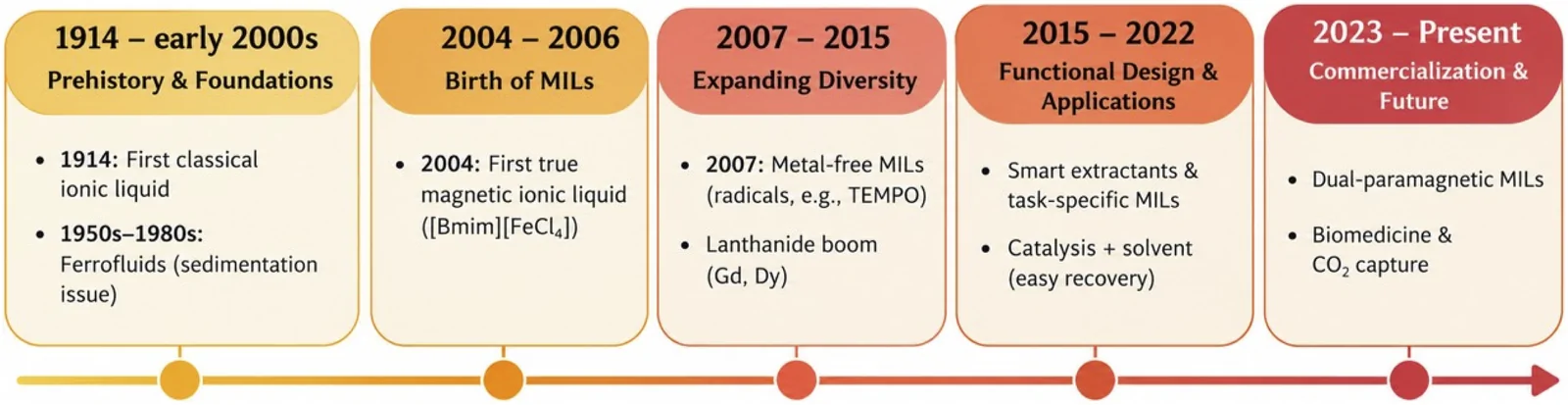
Key milestones in magnetic ionic liquid development.

A comparative summary of exemplary MIL-based systems' evolution, composition, design approaches, physicochemical characteristics, applications, and recovery traits is given in Table 5. It emphasizes how important characteristics like magnetic moment, viscosity, thermal stability, melting behavior, and hydrophobicity are influenced by incremental alterations, ranging from single magnetic centers and metal–ligand engineering to functionalization and dual-paramagnetic structures.^137^ While highlighting their recyclability and limitations, the table also links these structural and physicochemical changes with real-world uses, specifically magnetic extraction, separation, and functional magnetic liquids.^138^ Overall, it shows that rather than relying solely on magnetic strength, MIL performance requires on the simultaneous optimization of magnetic-center density, metal identity, ligand structure, functional-group position, and molecular architecture.

**Table 5:** Summary of synthesis strategy, composition, physicochemical properties, application, recyclability, and limitations of MILs

MIL-based system	Magnetic center/architecture	Synthesis or design strategy	Quantitative properties	Application	Performance/outcome	Main conclusion/limitation
[FeCl_4_]^−^-based MILs	Fe^3+^; single magnetic center	First-generation metal incorporation	*µ*_eff_ ≈ 5.66 µB; *χ*_MT_ = 3.49–4.50 emu mol^−1^ K Oe^−1^	Electrochemistry/magnetic liquids	Electron-transfer rate ≈ 3.60 × 10^−3^ cm^2^ s^−1^; diffusion coefficient ≈ 7.55 × 10^−1^ cm^2^ s^−1^	Established the basic concept of incorporating magnetic response directly into an IL; cation structure strongly affects properties^19^
Diglycolamide MILs	Transition metals/lanthanides	Metal–ligand engineering	*µ*_eff_ = 2.78–5.16 µB; viscosity as low as 198.3 cP; thermal stability up to 345 °C	Extraction/separation	Improved balance between magnetism and fluidity	Demonstrates that ligand engineering can reduce the magnetic-response/viscosity trade-off^21^
Lanthanide/transition-metal MILs	Gd^3+^, Dy^3+^, Ho^3+^, Er^3+^, Tb^3+^, Co^2+^, Ni^2+^, Mn^2+^, Fe^3+^	Coordination-environment engineering	*µ*_eff_ = 4.71–21.08 µB; transition-metal systems stable up to 235 °C; lanthanide systems ≈ 192 °C	Magnetic fluids/separation	Higher magnetic response than many first-generation systems	Increasing magnetic moment alone may increase density/viscosity; optimization must be multidimensional^2^
Co/Ni–[NTf_2_] MILs	Co^2+^/Ni^2+^	Metal substitution and ligand coordination	*µ*_eff_ = 2.78–5.16 µB; Co system ≈ 4.47 µB, Ni ≈ 2.78 µB	Functional magnetic liquids	Co substitution substantially increased magnetic response	Metal identity can have a stronger effect than ligand modification when spin state is maintained^21^
Functionalized diglycolate MILs	Transition/rare-earth metals	Cation/anion functionalization	Viscosity as low as 198.3 cP; thermal stability ≈ 345 °C	Extraction/separation	Improved fluidity and magnetic response	Functional-group location is as important as magnetic-ion identity^11^
[Ln(TODGA)_3_][Ln(hfac)_4_]_3_	Dual lanthanide centers	Fourth-generation dual-paramagnetic architecture	Magnetic response ≈ 4× comparable lanthanide-halide MILs	Magnetic extraction/functional liquids	Strong enhancement of magnetic response	Multiplying magnetic centers can be more effective than simply replacing one metal with another^30^
[Co(DMBG)_2_][Co(hfac)_3_]	Dual Co^2+^	Magnetic centers in both ions	Melting point = 341 K (68 °C); susceptibility ≈ 7.2 emu K mol^−1^	Hydrophobic extraction	Hydrophobic and magnetically recoverable	Dual-paramagnetic design increases magnetic functionality while retaining extraction capability^31^
[Mn(Cnim)_4_][Mn(hfac)_3_]_2_	Dual Mn^2+^	Dual-paramagnetic design	C_3_/C_6_ derivatives liquid at ambient conditions	Liquid–liquid microextraction	Strong hydrophobicity with liquid behavior	High magnetic-center density does not necessarily require sacrificing fluidity^32^
Oligonucleotide-functionalized MIL (OSMIL)	Magnetic MIL + oligonucleotide	Molecular recognition functionalization	Particle size ≈ 897 nm; magnetic collection ≈ 3 s	Sequence-specific DNA extraction	Selective DNA recognition and extraction	Demonstrates integration of molecular recognition with magnetic separation^33^
[P_66614_][FeCl_4_]	Fe^3+^	MIL-based microextraction	MIL volume = 75 µL	Hg extraction/speciation	Rapid magnetic phase separation without centrifugation	Reduces sample-processing steps and solvent volume^35^
MIL–carbon nanohorn/ICG system	Magnetic MIL + carbon nanohorn + indocyanine green	Multifunctional hybridization	Photothermal conversion efficiency = 63%; aqueous dispersion ≈ 7 days	Biomedical/theranostic	Magnetic guidance + fluorescence + photothermal activity	Shows potential for multi-stimulus biomedical platforms but requires extensive toxicity/biocompatibility validation^37^
[C_4_C_1_Im][FeCl_4_]	Fe^3+^	Solvent-free synthesis	Preparation ≈ 48 h	General MIL platform	Demonstrates solvent-free preparation	Solvent elimination is possible, but synthesis time remains relatively long^20^
Co/Ni-[NTf_2_] MILs, solvent-free	Co^2+^/Ni^2+^	One-pot heat/stir, solvent-free	Preparation generally ≈ 12 h, <50 °C; *µ*_eff_ = 2.78–5.16 µB; thermal stability up to 345 °C	Functional MIL synthesis	Some systems generated *in situ* in water <30 s	Strong evidence for simplified synthesis, although scalability and reproducibility require further validation^21^
Imidazolium/[FeCl_4_]^−^ MIL	Fe^3+^	Two-step synthesis	Electrochemical window ≈ 5.0 V; magnetic susceptibility ≈ 1.82 × 10^−1^ emu g^−1^	Electrochemistry	Stable electrochemical behavior	Demonstrates that relatively simple MIL preparation can retain useful electrochemical properties^54^
IL-functionalized MOF-808	IL/MOF composite	IL incorporation into coordination framework	Proton conductivity = 1.28 × 10^−1^ S cm^−1^ at 60 °C, 100% RH	Proton transport	Enhanced proton conduction	Combines IL tunability with framework stability^56^
IL@MIL-101(Cr)	Cr-MOF + IL	Coordination anchoring	Surface area = 1146 m^2^ g^−1^; propylene-carbonate production = 97.3%; selectivity = 100%; maintained activity for 5 cycles	Catalysis	High conversion/selectivity	Immobilization can prevent IL loss while maintaining catalytic activity^57^
Dicationic IL@MIL-101	IL/MOF	Ship-in-bottle confinement	BET area = 1162 m^2^ g^−1^; CO_2_ uptake = 47.4 cm^3^ g^−1^ at 273 K; propylene carbonate = 92.5%	CO_2_-related catalysis	High catalytic conversion	Porous confinement provides simultaneous control of porosity and ionic functionality^61^
Multi-SO_3_H IL@MIL-100(Fe)	Fe-MOF + acidic IL	IL immobilization	Acid sites = 0.9 mmol g^−1^; styrene-oxide conversion = 99% in 3 h	Catalysis	High conversion under solvent-free conditions	Strong acidic functionality with reduced active-component loss^62^
IL200@MOF-526	Fe-porphyrin + IL	Electronic-environment modification	CO production = 14.0 mmol g^−1^ in 72 h; selectivity = 97%	Photocatalytic CO_2_ conversion	Visible-light CO generation	Demonstrates electronic tuning of catalytic sites through IL incorporation^64^
Magnetic ionic-network nanoparticle	Fe_3_O_4_@SiO_2_ + poly(IL)	Sol–gel/immobilization	Catalyst loading = 0.1 mol%; ester yield = 78–99%; 10 cycles; 85 °C	Esterification	High yield with repeated use	Excellent example of magnetic separation combined with heterogeneous ionic-network catalysis^68^
Fe_3_O_4_@MCM-41–IL	Fe_3_O_4_ + silica + IL	One-pot sol–gel	BET area = 1177.4 m^2^ g^−1^; pore size ≈ 4.2 nm; pore volume = 1.80 cm^3^ g^−1^; *M*_s_ = 31.5 emu g^−1^	Magnetic micro-SPE	Pesticide recovery = 82.4–109.8%; LOD = 0.04–1.63 ng L^−1^	Combines mesoporosity, ionic functionality and magnetic separation effectively^65^
IL-functionalized Fe_3_O_4_ nanoparticles	Fe_3_O_4_ + IL	Microwave-assisted synthesis	*M*_s_ = 81 emu g^−1^ at 10 K and 73 emu g^−1^ at 300 K; particle size ≈ 6 nm	Magnetic nanomaterials	Superparamagnetic behavior; stable dispersion ≈ 3 months	Microwave synthesis enables rapid magnetic nanoparticle formation and surface functionalization^69^
[BIM][FeCl_4_]/[OIM][FeCl_4_]	Fe^3+^	Microwave-assisted heating	At 400 W/60 s: ≈ 81 and 93 °C at 0.1 wt%	Microwave-responsive systems	Rapid heating	Heating is mainly associated with ionic conductivity and dipolar polarization^72^
[BMIM]Cl FeCl_3_/[BMIM]Cl FeCl_2_	Fe^3+^/Fe^2+^	Electrochemical MIL system	Thermopower = 0.26 mV K^−1^; conductivity = 7 S m^−1^; charge-transfer resistance = 0.9 Ω cm^2^	Thermo-electrochemical energy	Magnetic field can influence Fe-containing species transport	Demonstrates integration of magnetic responsiveness with energy conversion^74^
[P_66614_]_2_[CoCl_4_]	Co^2+^	Biphasic extraction	Optimum MIL = 15 mg; NaCl = 7.5%; pH = 2; extraction = 60 min	PAH extraction	>99% pyrene extraction	Strong evidence for recyclable hydrophobic MIL extraction^76^
Bimetallic MIL microextraction system	Dual magnetic centers	Dual-functionalized MIL	MIL volume = 75 µL; extraction time = 5 min; enrichment factor >17; LOD = 7.50 ng L^−1^; inter-day RSD <11%	OH-PAH extraction	Rapid extraction and magnetic recovery	Miniaturization and magnetic recovery reduce processing requirements^78^
[C_4_mim][FeCl_4_]/[P_66614_][FeCl_4_]	Fe^3+^	Cation engineering	MIL densities generally 1.25–1.94 g cm^−3^; viscosities 21–65 mPa s; conductivity 0.23–1.37 S m^−1^	Transport/electrochemical applications	Properties strongly dependent on alkyl-chain length	Longer alkyl chains generally increase viscosity and decrease conductivity^90^
[P_66614_][FeCl_4_]	Fe^3+^	Phosphonium MIL	Viscosity ≈ 749 → 672 mPa s at 2 T	Magnetorheology	≈10.3% viscosity reduction under magnetic field	Demonstrates direct magnetic control of MIL flow properties^35^
[C_n_C_1_Im][FeCl_4_]	Fe^3+^	Alkyl-chain engineering	Density decreases 1.381 → 1.230 g mL^−1^ from C4 to C8; viscosity increases 45.8 → 60.9 mPa s	Magnetic fluid/transport	Chain length tunes density and viscosity	Hydrophobic-chain engineering provides a simple route to tune fluidity^20^
[C_n_C_1_Im][FeCl_3_Br]	Fe^3+^	Anion + alkyl-chain engineering	Viscosity increases 37.9 → 70.1 mPa s with chain extension	Magnetic fluids	Strong structure-dependent rheology	Cation-chain length is a major determinant of transport properties^20^
[C_2_mim][FeCl_4_]	Fe^3+^	Thermal/phase engineering	Solid–solid transition = 261 K; melting = 283 K; decomposition > 623 K	Thermal/phase applications	Broad thermal stability window	MILs may exhibit multiple phase transitions rather than a single melting point^2^
[3C_6_PC_14_][FeCl_4_]	Fe^3+^	Hydrophobic cation architecture	Thermal stability ≈ 623 K (350 °C) under N_2_	Thermal applications	Single-stage decomposition	Cation architecture strongly affects thermal operating range^83^
[AcMIm][FeCl_4_]	Fe^3+^	Acid-functionalized cation	Stable to ≈230 °C; degradation peaks ≈ 320, 482 and 597 °C	Acidic functional MIL	Multi-stage decomposition	Functionalization can substantially alter degradation pathways^84^
[C2mim][FeCl_4_] confined in SBA-15	Fe^3+^ + porous silica	Nanoscale confinement	Bulk melting = 287.5 K; confined melting = 235.4 K in 5.39 nm pores and 249.4 K in 8.36 nm pores	Confined MIL systems	Strong shift in phase-transition behavior	Nanoconfinement provides another method for controlling MIL phase behavior^86^
[Bmim][FeCl_4_]/PVDF hybrid	Fe^3+^	MIL/polymer hybrid	20 wt% MIL; C2 analogue magnetoionic coefficient = 1.06 ± 0.015 V cm^−1^ Oe^−1^	Magnetoionic materials	Cation chain length altered β-phase, porosity and magnetic response	Molecular design can translate directly into macroscopic device properties^8^
[Bmim][FeCl_4_]/P(VDF-TrFE)	Fe^3+^	Flexible polymer composite	40 wt% MIL; bending displacement = 2.1 mm at ∼3 kOe; magnetoelectric coefficient ≈ 15 V cm^−1^ Oe^−1^; sensitivity = 76 mV Oe^−1^	Sensors/actuators	Simultaneous magnetic sensing and actuation	Demonstrates multifunctional conversion of magnetic stimulus into electrical/mechanical response^119^
[N_1114_][NTf_2_] electrolyte	IL electrolyte	Electrolyte engineering	Specific capacitance ≈ 2000 F g^−1^ at 2.6 A g^−1^; stable operation up to 3.6 V	Supercapacitors	High-voltage energy storage	High electrochemical performance is promising, but viscosity/ion mobility remain constraints^122^
Li/LFP ionic-liquid electrolyte	Metal-containing IL/IL-type electrolyte	Electrolyte optimization	Coulombic efficiency = 96.3% after 250 cycles; capacity retention = 93.2% after 150 cycles	Li-ion energy storage	Stable cycling	Shows the importance of balancing conductivity, viscosity and electrochemical stability^121^
MIL-based DNA extraction system	Magnetic MIL + oligonucleotide	Recognition-functionalized MIL	Magnetic recovery ≈ 3 s; RSD = 1.19% (*n* = 3)	DNA/eDNA extraction	Selective extraction from mixed DNA/protein matrices	Demonstrates high selectivity combined with rapid magnetic phase manipulation^33^
Valine-derived MIL surfactant/gelatin hydrogel	Magnetic IL surfactant	MIL/polymer hydrogel	Encapsulation efficiency: 69 ± 0.6% for ornidazole; 78 ± 0.3% for 5-FU	Drug delivery	Magnetic-responsive encapsulation	MILs can act as functional components in stimuli-responsive biomedical systems^126^
[P_66614_]_2_[CoCl_4_]/[P_66614_]_3_[DyCl_6_]	Co^2+^/Dy^3+^	Hydrophobic MIL extraction phases	Co-MIL: optimum 15 mg, 7.5% NaCl, pH 2	PAH extraction	>99% pyrene extraction	Co-based system retained high extraction efficiency during repeated use^76^
[N_1,8,8,8_][FeCl_4_]	Fe^3+^	Magnetic microextraction	MIL = 3.3 mg mL^−1^; extraction = 30 min	Cd/Zn extraction	99.7% Cd and 81.0% Zn separation	Demonstrates selective metal extraction with simplified phase separation^36^
[C_16_mim][FeCl_4_] microemulsion	Fe^3+^	MIL-based microemulsion	Residual oil = 1.36% after treatment	Oily-sludge remediation	High oil removal	Indicates potential for magnetic treatment of complex oily waste streams^128^
Silica-immobilized magnetic dicationic IL/FeCl_4_^−^	Fe^3+^	Immobilized dicationic MIL	CO_2_ uptake = 57.31 ± 0.02 mg g^−1^ at 1 bar, 25 °C	CO_2_ capture	CO_2_/N_2_ selectivity ≈ 3.8× that of pure silica	Combines magnetic functionality, gas capture and easier phase recovery^129^
[C_4_C_1_im][FeCl_4_]	Fe^3+^	Magnetic-field-responsive MIL	Viscosity decrease = 10.3% at 2 T	Smart fluids/microfluidics	Field-controlled flow	Magnetic field can regulate fluid resistance without mechanical components^118^
[C_8_C_1_im][FeCl_4_]	Fe^3+^	Longer-chain MIL	Viscosity decrease = 20.1% at 2 T	Smart fluids	Greater field-induced viscosity changes than shorter-chain analogue	Alkyl-chain architecture can tune magnetic-field sensitivity^118^
[Bmim][FeCl_4_] + Fe_3_O_4_/Fe_2_O_3_	Fe^3+^ MIL + magnetic nanoparticles	MIL/nanoparticle hybrid	Field-induced viscosity/relaxation changes; shear-thinning behavior	Magnetorheological fluids	Transition toward more elastic field-responsive behavior	Nanoparticle incorporation increases magnetic responsiveness but introduces hybrid-system complexity^80^
[Bmim][FeCl_4_] in CF10W-40	Fe^3+^	MIL lubricant additive	MIL = 2 wt%; wear depth ↓45%; friction ↓∼20%; at 0.1 T further wear ↓20%, friction ↓∼10%; critical load ↑∼20% at 1 T	Tribology	Magnetically controlled lubrication	Demonstrates that MILs can provide both lubrication and magnetic control^131^
TEMPO-derived metal-free MILs	Organic radical	Metal-free radical design	C_2_ derivative melting point ≈ 330.15 K (57 °C); C_4_/C_6_/C_8_ derivatives liquid at ambient conditions	Magnetic liquids/separation	Magnetic response without metal centers	Reduces dependence on transition/rare-earth metals, but radical stability remains an important issue^40^

## 5. Challenges and future outlook

Despite their adaptability, safety, cost, scalability, and regulatory ambiguity continue to be barriers to MILs' transfer from lab systems to useful technology. Future designs must prioritize biodegradability, low-toxicity components, metal-leaching management, and life-cycle evaluation because their low volatility does not always indicate environmental benignity because toxicity can result from certain cations, anions, or metal centers. However, commercialization is hampered by costly precursors, multistep synthesis, and restricted large-scale processing, underscoring the need for one-pot, solvent-free, inexpensive, continuously scalable synthesis with proven long-term recyclability.^139^

Experimental evidence confirms that MIL toxicity depends on both the cation structure and the metal-containing anion. Sintra *et al.* evaluated cholinium-based MILs using the luminescent bacterium *Vibrio fischeri* and found that all tested structures were moderately toxic or toxic. Toxicity varied with alkyl side-chain length, the number of hydroxyethyl groups, and the atomic number of the metal in the anion; among the magnetic anions investigated, [MnCl_4_]^2−^ exhibited the lowest toxicity.^101^ Furthermore, Frade *et al.* demonstrated that the metal-containing anions [CoCl_4_]^2−^ and [MnCl_4_]^2−^ were more prone to induce cytotoxicity in human skin fibroblasts and colorectal adenocarcinoma cell lines than other tested systems. These findings indicate that environmental and biological safety must be evaluated for individual MIL formulations rather than inferred from their negligible vapour pressure.^101^

Metal leaching is another important concern for MIL-based extraction and environmental remediation. The release of transition-metal or lanthanide species into treated water during extraction, regeneration, or disposal could introduce secondary contamination and compromise material stability. Therefore, future investigations should quantify dissolved metal concentrations using inductively coupled plasma mass spectrometry (ICP-MS) or inductively coupled plasma optical emission spectroscopy (ICP-OES). Metal-leaching percentages, residual metal concentrations, and changes in MIL composition should be reported alongside extraction efficiency. The absence of sufficient long-term leaching and environmental-fate data remains an important limitation in establishing the suitability of MILs for large-scale water treatment.

Progress towards automated processing has also been demonstrated by Will *et al.*, who developed an *in situ* MIL-generation method coupled with parallel-dispersive droplet extraction. Their 96-well platform enabled simultaneous processing of up to 96 samples, with a reported sample-preparation time of 0.78 min per sample. However, relative recoveries varied from 53.9% to 129.1% across the investigated analytes, indicating that analytical performance can remain compound-dependent even when processing throughput is improved. This example illustrates the distinction between high-throughput laboratory automation and industrial-scale manufacturing or continuous environmental treatment.^97^ Consequently, one-pot or solvent-free synthesis, inexpensive precursors, reduced purification requirements, and continuously scalable production methods should be prioritized.

The lack of defined procedures for assessing magnetic characteristics, extraction efficiency, toxicity, leaching, regeneration, and stability further hinders progress and makes it challenging to compare MIL systems. In order to anticipate structure–property interactions and expedite safer MIL design, it will be necessary to integrate DFT, molecular dynamics, machine learning, materials chemistry, and environmental engineering more closely. Therefore, creating fewer, safer, recyclable, scalable, and application-validated materials that can close the gap between laboratory performance and practical environmental applications is more important for the future than just creating new MIL compositions.^140^

Translating MIL-based technologies from laboratory synthesis to pilot-scale deployment remains challenging due to the need for reproducible large-batch synthesis, cost-effective purification, and consistent material properties. Practical implementation also requires evaluating MIL recovery and regeneration efficiency, metal leaching, long-term stability, and performance in real-world matrices containing competing ions and organic matter. Furthermore, continuous-process integration, waste management, equipment compatibility, and techno-economic and life-cycle assessments are essential to establish operational feasibility. Pilot-scale studies under realistic operating conditions are therefore needed to validate scalability, environmental safety, and cost-effectiveness before commercial application.^141^

## 6. Conclusions

From straightforward magnetically responsive liquids, MILs are quickly developing into adaptable, multifunctional platforms with enormous potential in sensing, environmental remediation, extraction, catalysis, separation, and energy-related applications. Recent studies show that magnetic responsiveness, viscosity, stability, and application-specific functionality can be efficiently tailored using metal–ligand engineering, bimetallic structures, immobilization techniques, and magnetic nanocomposites. Finding a balanced combination of magnetism, fluidity, chemical stability, low toxicity, recyclability, and economic viability is still a significant task, though. Therefore, future studies should concentrate on structure–property interactions, standardized stability evaluation, leaching and toxicity, life-cycle assessment, and scalable synthesis instead of maximizing magnetic susceptibility. The creation of safer and task-specific MILs may be accelerated by combining computational modeling and machine learning with experimental design. Transforming MILs from promising laboratory materials into sustainable and actually deployable next-generation functional technologies will require addressing these issues.

## Author contributions

Writing – original draft, and writing – review & editing, H. S. S., N. K., and N. K.; investigation, and conceptualization, M. K., H. H. and N. B.; supervision, H. S. S., and P. K. B. All authors have read and agreed to the published version of the manuscript.

## Conflicts of interest

The author declares no competing interests.

## Data Availability

Data availability is not applicable to this article as no new data were created or analyzed in this study.
